# Identification of aberrantly methylated differentially expressed genes and associated pathways in endometrial cancer using integrated bioinformatic analysis

**DOI:** 10.1002/cam4.2956

**Published:** 2020-03-14

**Authors:** JinHui Liu, YiCong Wan, Siyue Li, HuaiDe Qiu, Yi Jiang, Xiaoling Ma, ShuLin Zhou, WenJun Cheng

**Affiliations:** ^1^ Department of Gynecology The First Affiliated Hospital of Nanjing Medical University Nanjing China; ^2^ Center of Rehabilitation Medicine The First Affiliated Hospital of Nanjing Medical University Nanjing China

**Keywords:** bioinformatic analysis, CMap, endometrial cancer, gene expression omnibus (GEO), methylation, PPI, risk model

## Abstract

Endometrial cancer (EC) is a fatal female reproductive tumor. Bioinformatic tools are increasingly developed to screen out molecular targets related to EC. In this study, http://www.ncbi.nlm.nih.gov/geo/query/acc.cgi?acc=GSE17025 and http://www.ncbi.nlm.nih.gov/geo/query/acc.cgi?acc=GSE40032 were obtained from Gene Expression Omnibus (GEO). “limma” package and Venn diagram tool were used to identify hub genes. FunRich was used for functional analysis. Retrieval of Interacting Genes Database (STRING) was used to analyze protein‐protein interaction (PPI) complex. Cancer Genome Atlas (TCGA), GEPIA, immunohistochemistry staining, and ROC curve analysis were carried out for validation. Univariate and multivariate regression analyses were performed to predict the risk score. Compound muscle action potential (CMap) was used to find potential drugs. GSEA was also done. We retrieved seven oncogenes which were upregulated and hypomethylated and 12 tumor suppressor genes (TSGs) which were downregulated and hypermethylated. The upregulated and hypomethylated genes were strikingly enriched in term “immune response” while the downregulated and hypermethylated genes were mainly focused on term “aromatic compound catabolic process.” TCGA and GEPIA were used to screen out *EDNRB, CDO1, NDN, PLCD1, ROR2, ESPL1, PRAME, and PTTG1*. Among them, *ESPL1* and *ROR2* were identified by Cox regression analysis and were used to construct prognostic risk model. The result showed that *ESPL1* was a negative independent prognostic factor. Cmap identified aminoglutethimide, luteolin, sulfadimethoxine, and maprotiline had correlation with EC. GSEA results showed that “hedgehog signaling pathway” was enriched. This research inferred potential aberrantly methylated DEGs and dysregulated pathways may participate in EC development and firstly reported eight hub genes, including *EDNRB, CDO1, NDN, PLCD1, ROR2, ESPL1, PRAME,* and *PTTG1* that could be used to predict EC prognosis. Aminoglutethimide and luteolin may be used to fight against EC.

## Graphical abstract

This research inferred potential aberrantly methylated DEGs and dysregulated pathways may participate in EC development and firstly reported 8 hub genes, including EDNRB, CDO1, NDN, PLCD1, ROR2, ESPL1, PRAME and PTTG1 that could be used to predict EC prognosis. Aminoglutethimide and luteolin may be used to fight against EC.

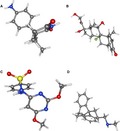

**Abbreviations**
CI: confidence interval
CMap: compound muscle action potential
DEGs: differentially expressed genes
DMGs: differentially methylated genes
EC: Endometrial cancer
GEO: gene expression omnibus
GO: Gene Ontology
GSEA: Gene set enrichment analysis
KEGG: Kyoto Encyclopedia of Genes and Genomes
PPI: protein‐protein interaction
ROC: Receiver operating characteristic
STRING: Search Tool for the Retrieval of Interacting Genes Database
TCGA: the Cancer Genome Atlas
TSGs: tumor suppressor genes

## 1 BACKGROUND

Originating from endometrial cells, endometrial cancer (EC) is a lethal tumor in female reproductive system.^1^ In 2015, the American Cancer Society (ACS) predicted that the number of new cases of EC was 54 870, and 10 170 of them had died. This means that in the past 20 years, the mortality of EC has almost doubled. The average age at the establishment of EC diagnosis is 63. EC is most likely to attack postmenopausal women, 90% of whom are over 50 years old.^2^ The symptoms of EC, represented by irregular vaginal bleeding after menopause, are often overlooked, making early EC diagnosis a thorny challenge.^3^ To solve this challenge, database‐based bioinformatic analysis has been increasingly used to screen out target biological molecules of diagnostic value.^4^ For example, Shenghui Yao et al screened out some DEGs causing cervical intraepithelial neoplasia via GEO database.^5^ Xiangsheng Liu et al analyzed the gene modules related to human osteosarcoma through a coexpression network.^6^ DNA methylation of the gene promoter region typically inhibits gene expression. Most methylated CpG islands are located within genes or intergenic regions, while less than 3% in the gene promoter region. Intra‐ or intergene DNA methylation may regulate gene expression.^7^ Many studies in recent years have shown that DNA methylation is closely related to tumor progression.^8^, ^9^, ^10^ Extensive research has found that DNA methylation affects the occurrence and progression of EC.^11^, ^12^, ^13^, ^14^ We reasonably speculated that some methylation genes act on EC and could be used as biomarkers for targeted therapy of EC. In this research, we first screened out DEGs from http://www.ncbi.nlm.nih.gov/geo/query/acc.cgi?acc=GSE17025, and hypermethylated/hypomethylated genes from http://www.ncbi.nlm.nih.gov/geo/query/acc.cgi?acc=GSE40032. Comprehensive analysis, functional analysis, and PPI network analysis were performed. Potential drugs, hub genes, and terms related to EC were determined.

## 2 MATERIALS AND METHODS

### 2.1 Microarray data profile

The study design is shown in Figure 4A. Gene Expression Omnibus (GEO) is an online database which provides comprehensive data on gene profiling and sequencing. In GEO database (https://www.ncbi.nlm.nih.gov/geo/), we retrieved the gene expression profile dataset http://www.ncbi.nlm.nih.gov/geo/query/acc.cgi?acc=GSE17025 along with methylation profile dataset http://www.ncbi.nlm.nih.gov/geo/query/acc.cgi?acc=GSE40032. Based on the GPL570 platform [HG‐U133_Plus_2] Affymetrix Human Genome U133 Plus 2.0 Array,^15^ we collected data from http://www.ncbi.nlm.nih.gov/geo/query/acc.cgi?acc=GSE17025 containing 91 samples of stage I endometrial cancers and 12 samples of postmenopausal atrophic endometrium. Based on GPL8490, we collected the methylation profile microarray data from http://www.ncbi.nlm.nih.gov/geo/query/acc.cgi?acc=GSE40032, including 64 endometrial cancer samples and 23 cancer‐free samples.^16^

### 2.2 Data processing and identification of DEGS and DMGS

To explore the differentially expressed genes (DEGs) and differentially methylated genes (DMGs), we applied the “limma” packages^17^ for processing http://www.ncbi.nlm.nih.gov/geo/query/acc.cgi?acc=GSE17025 and http://www.ncbi.nlm.nih.gov/geo/query/acc.cgi?acc=GSE40032 datasets. The DEGs were screened with criteria |logFC| > 1 and adj‐*P*‐value < .05, while DMGs were identified with FDR < 0.05 and |logFC| > 0.2. We reviewed previously published literature before setting criteria.^18^, ^19^, ^20^ Subsequently, oncogene and TSG lists were teased out of two online databases (http://ongene.bioinfo-minzhao.org/, http://bioinfo.uth.edu/TSGene/index.html), respectively. To illustrate the intersection among DEGs, DMGs, oncogenes, and TSGs, an Venn diagram program^21^ was employed. As a result, upregulated hypomethylated oncogenes as well as downregulated hypermethylated genes were filtered out.

### 2.3 Pathway analysis of DEGS

Gene Ontology (GO) function and Kyoto Encyclopedia of Genes and Genomes (KEGG) pathways involving the two gene lists were determined using an enrichment analysis online tool FunRich.^22^ The biological function of the overlap genes was interpreted. A value of *P* < .05 was seen to be statistically significant.

### 2.4 PPI network construction

STRING^23^ and FunRich were used to build PPI network that hinted at the molecular mechanism involved in EC tumorigenesis. The protein‐protein interaction (PPI) network was plotted by Cytoscape v3.7.0. A value of *P* < .05 was considered statistically significant.

### 2.5 Validation of the selected genes

To validate the aberrantly methylated DMGs, the data from the Cancer Genome Atlas (TCGA) database were analyzed using the online software GEPIA (http://gepia.cancer-pku.cn/)^24^ for cancer and healthy gene expression profiles. In order to confirm epigenetic methylation level, we validated using TCGA data and R software. We analyzed the complete follow‐up data of all EC patients. To validate the selected oncogenes and TSGs on the translational level, the immunohistochemistry stained samples of both the normal and endometrial cancer were downloaded from the Human Protein Atlas database (https://www.proteinatlas.org/). ROC curve analysis was for distinguishing normal and cancer tissues.

### 2.6 Construction of a prognostic signature

First, univariate Cox proportional hazards regression analysis was performed based on eight genes. Prognosis‐associated genes (*P* < .05) were determined. Next, multivariate Cox regression analysis was utilized to identify significant prognosis‐related genes. Regression model was constructed to assess each patient's risk score for gene expression. Patients were divided into low‐ and high‐risk groups based on the average risk score. Then, we used Kaplan‐Meier curve analysis to compare the survival time of the low‐ and the high‐risk group. Cutoff was set as *P* < .05. In addition, we used the receiver operating characteristic (ROC) curve to predict the 5‐year survival. The predictive value was calculated as the area under the curve, sensitivity, and specificity. Harrel's concordance index (*C‐index*) was measured to validate the predictive ability of this signature using the “survcomp” R package.^25^

### 2.7 Identification of candidate small molecules

We used CMap to find potential drugs related to EC. CMap is a program for predicting potential drugs that may induce biological status encoded by specific gene expression signatures.^26^ Upregulated hypomethylated genes and downregulated hypermethylated genes were used to query the CMap database. Finally, the enrichment score representing similarity was calculated, ranging from −1 to 1. The positive connectivity score indicates that drugs can induce the biological phenomena queried in human cell lines. Conversely, a negative connectivity score indicates that the drug reverses the requested biological characteristics and has potential therapeutic value. The connectivity scores (*P* < .05) for the various instances were filtered out. Tomographic scans of these potential effective drugs were studied in Pubchem.

### 2.8 Gene set enrichment analysis (GSEA)

In order to reveal the function of eight key genes, GSEA (http://software.broadinstitute.org/gsea/index.jsp)^27^ was used to determine the enrichment of previously defined biological processes in the ranked DEGs between the two groups.^3^ From TCGA, 546 EC samples were divided into two different groups based on their median expression levels. The collection of annotated gene sets of c2.cp.kegg.v6.0.symbols.gmt in Molecular Signatures Database (MSigDB, http://software.broadinstitute.org/gsea/msigdb/index.jsp) was chosen as the reference gene sets in GSEA software, and the *P*‐value < .05 was set as the cutoff.

## 3 RESULTS

### 3.1 DEGs and DMGs in endometrial cancers

**FIGURE 1:**
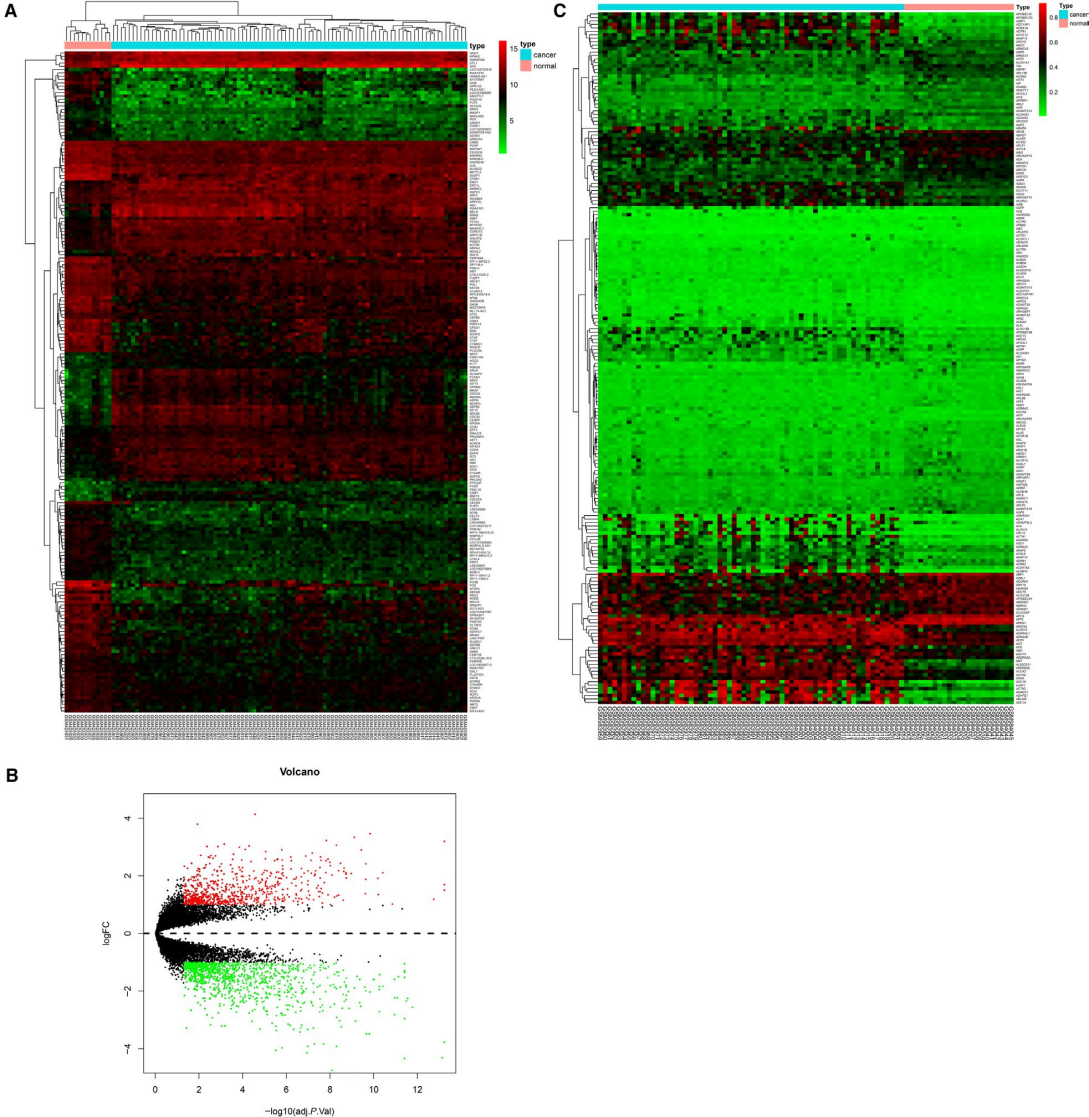
DEGs in http://www.ncbi.nlm.nih.gov/geo/query/acc.cgi?acc=GSE17025 and DMGs in http://www.ncbi.nlm.nih.gov/geo/query/acc.cgi?acc=GSE40032. A, Volcano Plot visualizing all the DEGs. Red dots represent upregulated genes, green dots represent downregulated genes, and black dots represent genes without differential expression. B, Heatmap of the top 200 DEGs. C, Heat map of the top 200 DMGs

Expression matrices were obtained from http://www.ncbi.nlm.nih.gov/geo/query/acc.cgi?acc=GSE17025 consisting of 91 endometrial cancer, 12 serous samples of atrophic endometrium from postmenopausal women. The DEGs were presented in Figure 1A,B. We obtained 1737 DEGs, including 690 upregulated and 1047 downregulated. A total of 4097 DMGs were obtained in http://www.ncbi.nlm.nih.gov/geo/query/acc.cgi?acc=GSE40032 (Figure 1C). As a result, 1761 hypermethylated genes and 2336 hypomethylated genes were also screened out.

### 3.2 Aberrantly methylated DEGS

**FIGURE 2:**
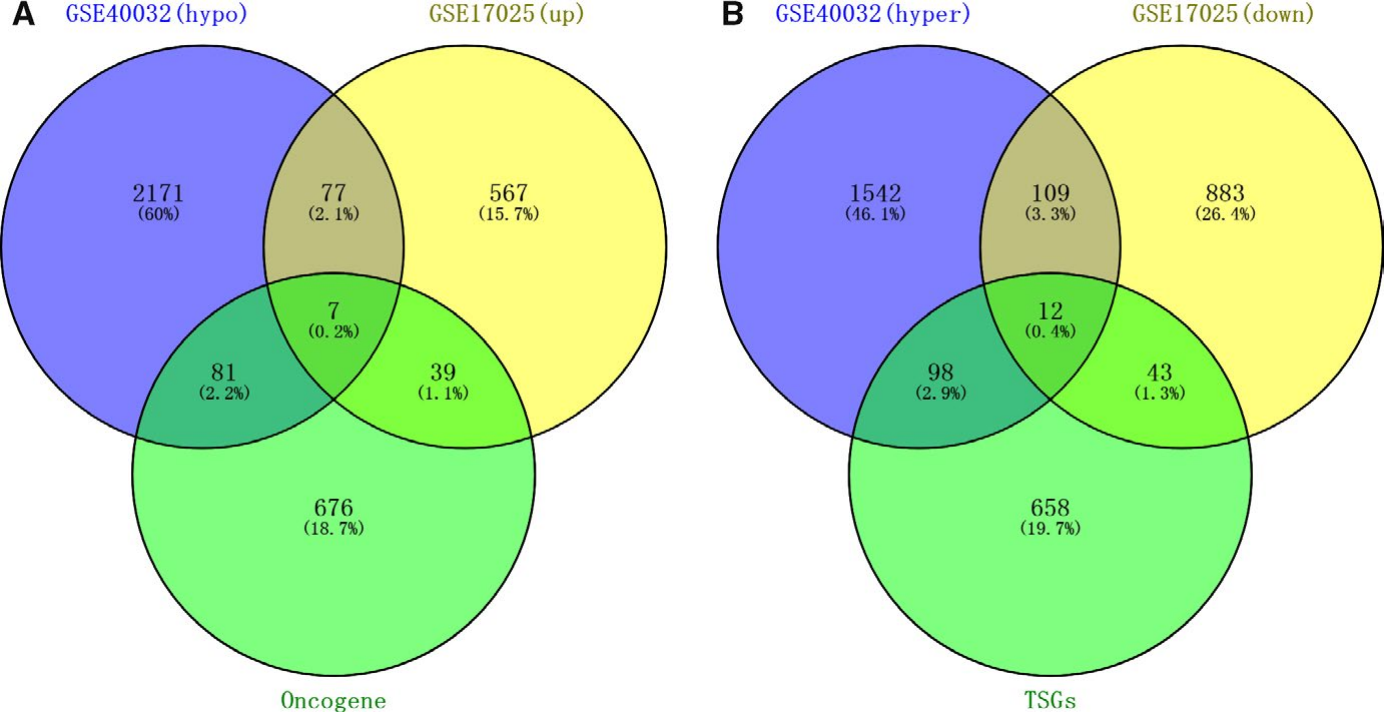
Aberrantly methylated DEGs, and associated oncogenes and tumor suppressor genes (TSGs). A, 84 hypomethylated and upregulated genes were identified, including seven oncogenes. B, 121 hypermethylated and downregulated genes were identified, including 12 TSGs

Through Venn analysis, we identified the genes with overlapped expression between the upregulated genes and hypomethylated genes. We also identified the genes with overlapped expression between downregulated genes and hypermethylated genes. Subsequently, we searched 121 downregulated hypermethylated genes and 84 upregulated hypomethylated genes. So as to pinpoint the aberrantly methylated DEGs, upregulated hypomethylated genes were overlapped with oncogenes, and downregulated hypermethylated genes with TSGs. Correspondingly, we retrieved seven upregulated hypomethylated oncogenes (Figure 2A). We also identified 12 downregulated hypermethylated TSGs, indicating hypomethylation may downregulate the expression of these specific genes in tumorigenesis (Figure 2B).

### 3.3 Go and KEGG pathway analysis of DEGS

**FIGURE 3:**
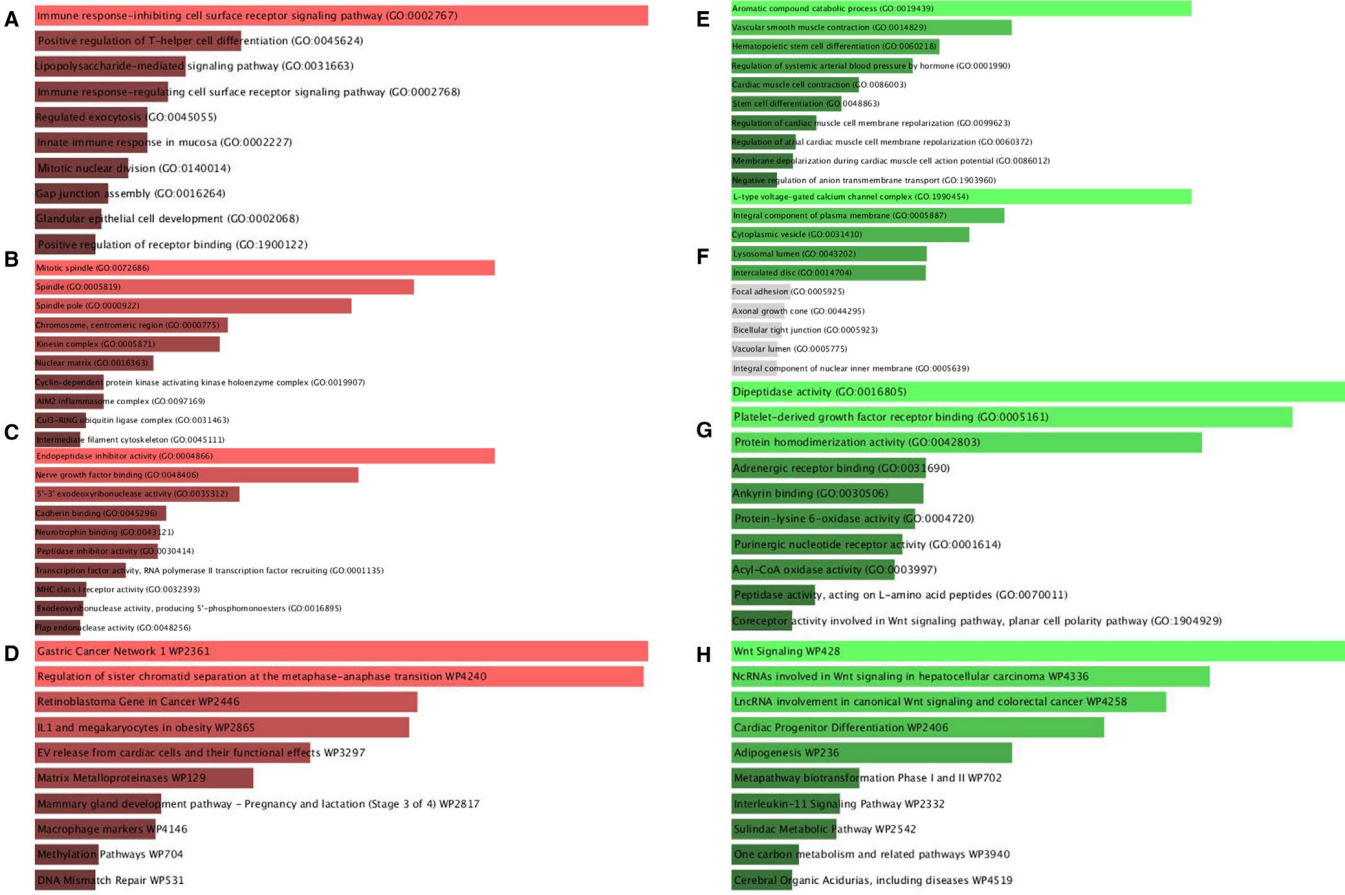
GO functional annotation of aberrantly methylated DEGs. A, The top 10 enriched BP items of upregulated hypomethylated genes B, the top 10 enriched CC items of upregulated hypomethylated genes; C, the top 10 enriched MF items of upregulated hypomethylated genes; D, the top 10 enriched pathways items of upregulated hypomethylated genes; E, the top 10 enriched BP items of downregulated hypermethylated genes (hypermethylated, lowly expressed genes); F, the top 10 enriched CC items of downregulated hypermethylated genes; G, the top 10 enriched MF items of downregulated hypermethylated genes. H, the top 10 enriched pathways of downregulated hypermethylated genes

GO terms cover biological process (BP), molecular function (MF), and cellular component (CC) ontologies. In our study, BP terms of upregulated hypomethylated genes were significantly enriched in immune response, positive regulation of T‐helper cell differentiation, and lipopolysaccharide‐mediated pathway (Figure 3A). CC terms of upregulated hypomethylated genes were significantly enriched in mitotic spindle, spindle pole, and chromosome in the centrometric region (Figure 3B). MF terms of the genes were mostly enriched in endopeptidase inhibitor activity, nerve growth factor binding, and 5′‐3′ exodeoxyribonuclease (Figure 3C). KEGG analysis indicated that these genes were mainly involved in Gastric Cancer Network 1, regulation of sister chromatid separation at the metaphase‐anaphase transition as well as retinoblastoma gene in cancer (Figure 3D). By contrast, downregulated hypermethylated genes were mainly enriched in: (a) aromatic compound catabolic process, vascular smooth muscle contraction, and hematopoietic stem cell differentiation (BP, Figure 3E); (b) L‐type voltage‐gated calcium channel complex, integral component of plasma membrane, and cytoplasmic vesicle (CC, Figure 3F); (c) dipeptidase activity, platelet‐derived growth factor receptor binding, and protein homodimerization activity (MF, Figure 3G). These genes were mainly enriched in term “Wnt signaling pathway” (Figure 3H).

### 3.4 PPI network construction

**FIGURE 4:**
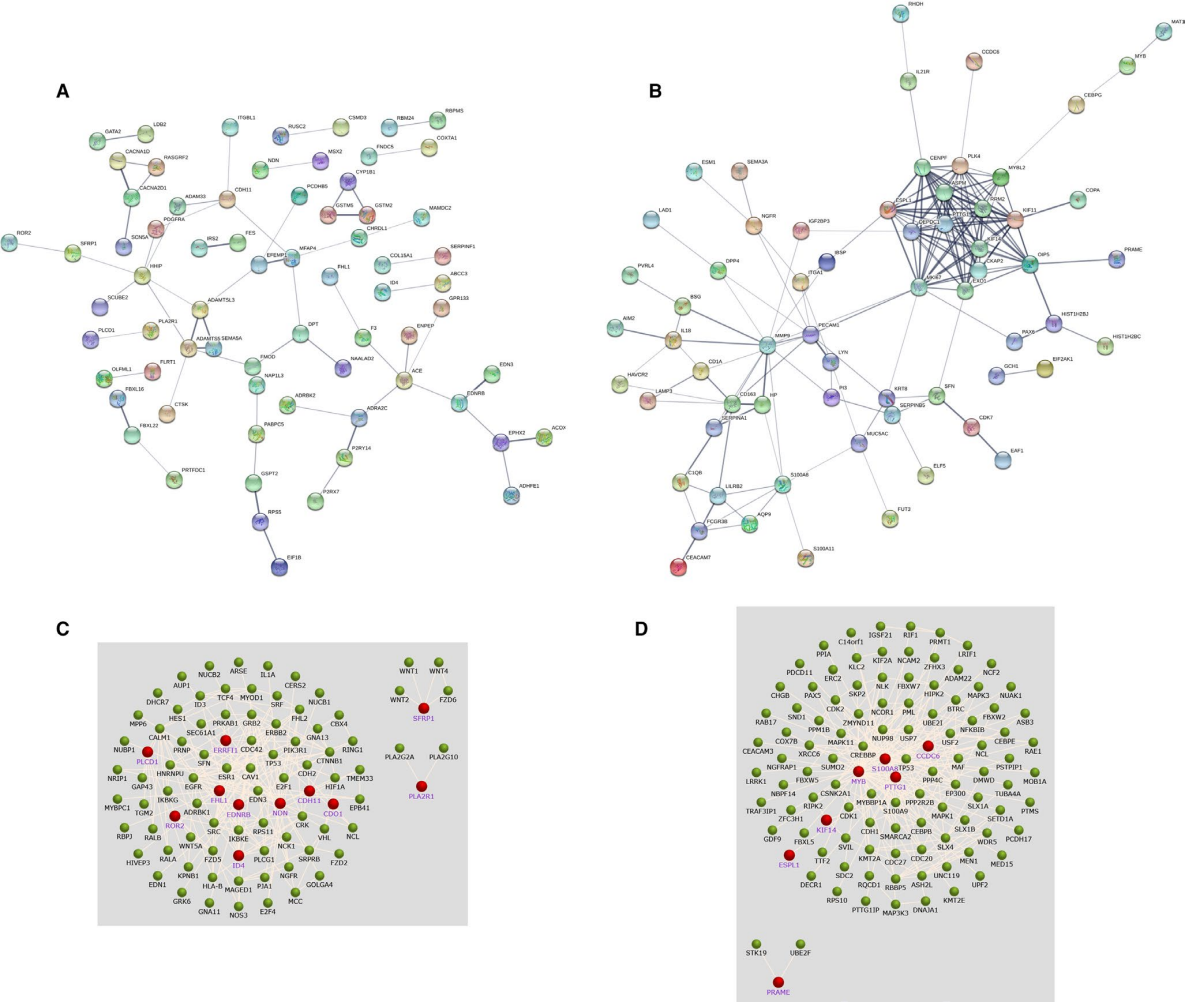
PPI network for the aberrantly methylated DEGs. A, 121 genes filtered into the downregulated hypermethylated PPI network containing 120 nodes and 60 edges. B, 84 genes filtered into the upregulated hypomethylated PPI network containing 84 nodes and 164 edges. C, The PPI network of 11 downregulated hypermethylated TSGs, and their related genes, created by the FunRich software. D, The PPI network of the seven upregulated hypomethylated oncogenes, and their related genes, created by the FunRich software

STRING was for constructing the PPI network. A total of 120 nodes and 60 edges were involved in the PPI network of the downregulated hypermethylated genes, with a PPI enrichment *P*‐value of 3.88e‐05 (Figure 4A). PPI complex of the upregulated hypomethylated genes consisted of 84 nodes and 164 edges (*P*‐value: 1.0e‐16) (Figure 4B). A total of 11 downregulated hypermethylated TSGs and seven upregulated hypomethylated oncogenes along with their associated genes are presented in Figure 4C,D. The pathways involving the 11 downregulated hypermethylated TSGs and their associated genes are shown in Table S1. These genes were mostly enriched in “pathway in cancer.” The pathways involving the seven upregulated hypomethylated oncogenes and their associated genes are shown in Table S2. These genes were mostly enriched in “cell cycle.”

### 3.5 Validation of the selected genes

**FIGURE 5:**
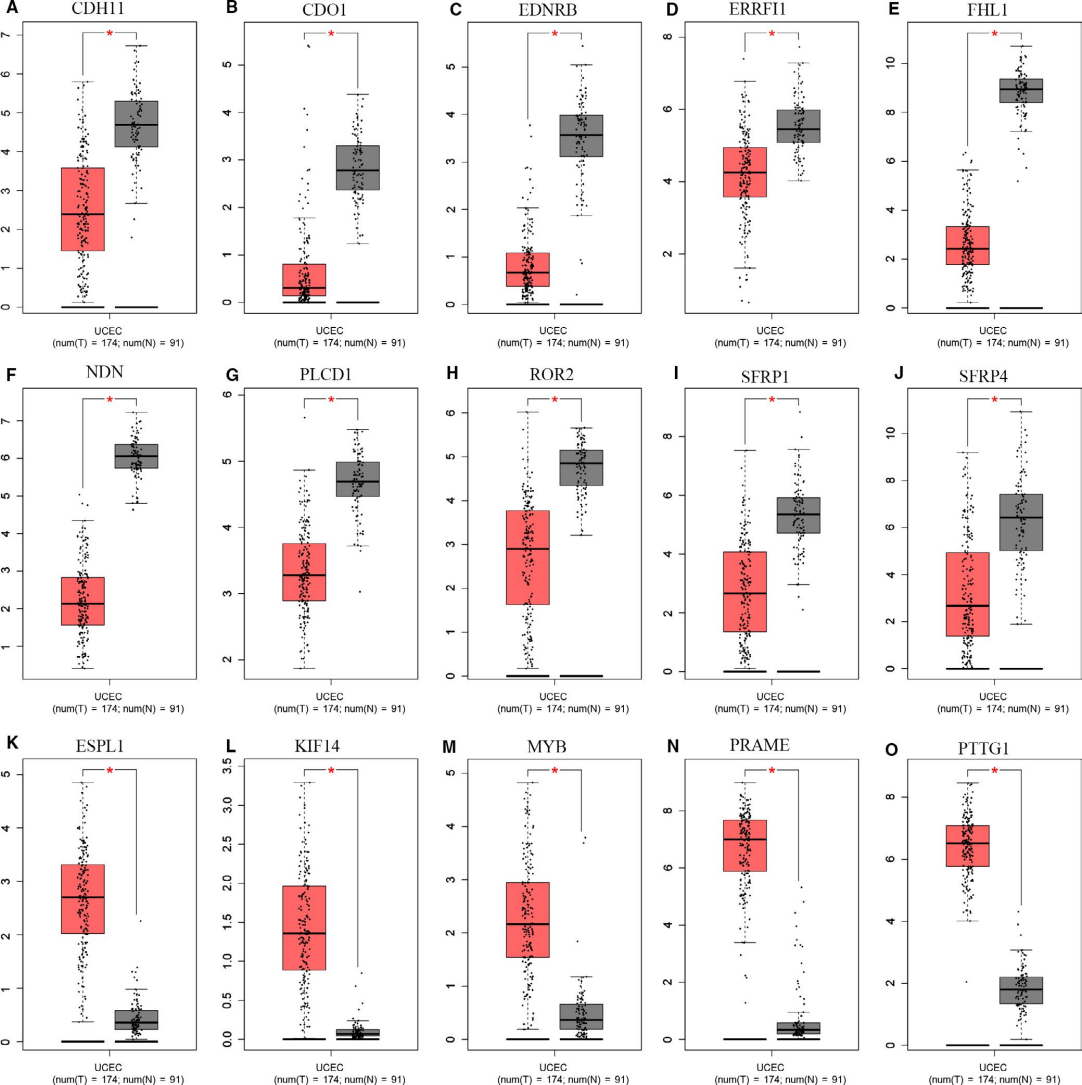
Validation of the 19 genes in the GEPIA. A‐D, Box plots showing the expression of the 15 genes was the same to that in our study based on GEPIA (*P*‐values < 0.05). The red node represented tumor samples, gray node represented normal samples

**FIGURE 6:**
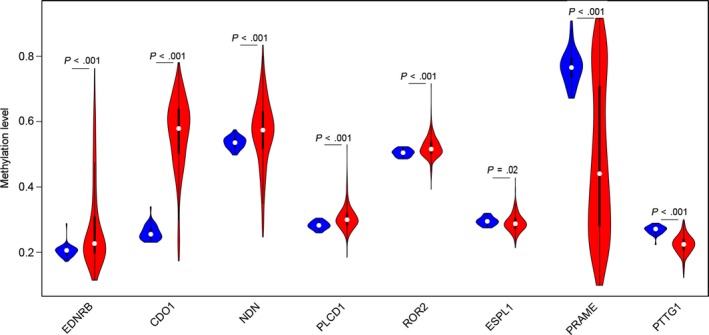
Validation of the 15 genes in the TCGA database. Violin plots showing the methylation status of the eight genes similar to that in our study based on TCGA database. The red violin represented the tumor samples and the blue violin represented the healthy samples

**FIGURE 7:**
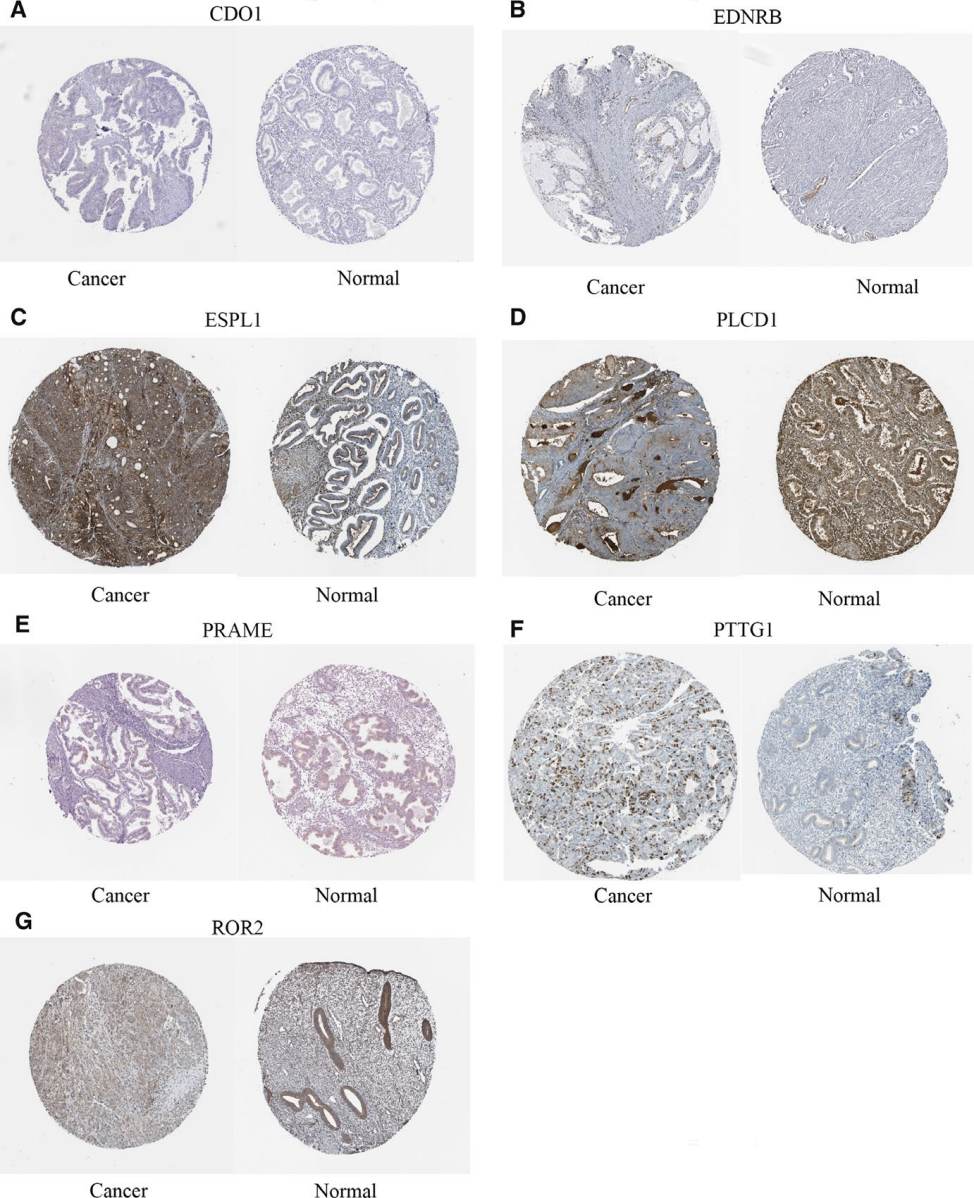
Immunohistochemistry (IHC) of the seven genes based on The Human Protein Atlas. A, Protein levels of CDO1 in tumor tissue (staining: negative; intensity: negative; quantity: none). Protein levels of CDO1 in normal tissue (staining: negative; intensity: negative; quantity: none). B, Protein levels of EDNRB in tumor tissue (staining: negative; intensity: negative; quantity: none). Protein levels of EDNRB in normal tissue (staining: medium; intensity: moderate; quantity: 75%‐25%). C, Protein levels of *ESPL1* in tumor tissue (staining: high; intensity: strong; quantity: >75%). Protein levels of *ESPL1* in normal tissue (staining: high; intensity: strong; quantity: 75%‐25%). D, Protein levels of PLCD1 in tumor tissue (staining: medium; intensity: moderate; quantity: 75%‐25%). Protein levels of PLCD1 in normal tissue (staining: high; intensity: strong; quantity: >75%). E, Protein levels of PRAME in tumor tissue (staining: low; intensity: moderate; quantity: <25%). Protein levels of PRAME in normal tissue (staining: negative; intensity: negative; quantity: none). F, Protein levels of PTTG1 in tumor tissue (staining: high; intensity: strong; quantity: <25%). Protein levels of PTTG1 in normal tissue (staining: medium; intensity: strong; quantity: <25%). G, Protein levels of ROR2 in tumor tissue (staining: low; intensity: weak; quantity: >75%). Protein levels of ROR2 in normal tissue (staining: medium; intensity: moderate; quantity: >75%)

**FIGURE 8:**
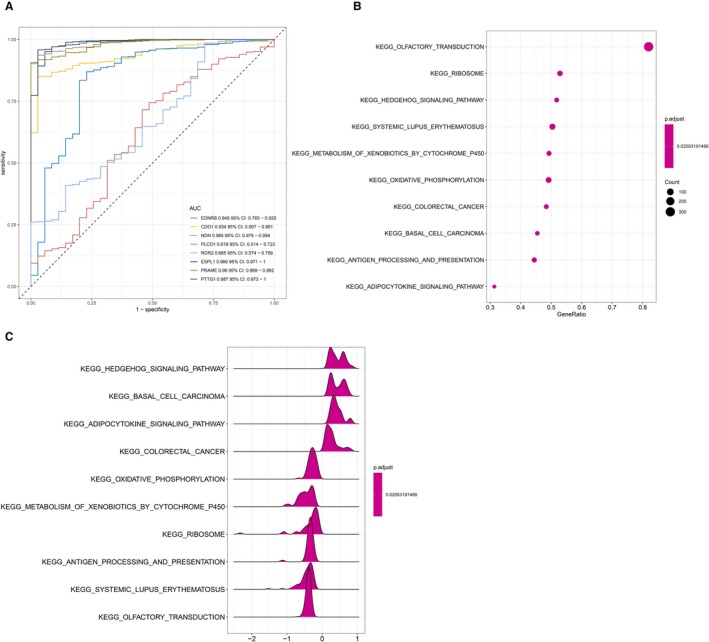
A, ROC curve analysis and AUC analysis were implemented to evaluate the capacity of eight genes to distinguish EC tissue from normal tissue. B‐C, GSEA using TCGA UCEC databases. The 10 most functional gene sets enriched in EC samples

We used the data from TCGA to confirm how the expression and methylation of selected genes work in EC carcinogenesis. Five upregulated hypomethylated oncogenes, along with 10 downregulated hypermethylated TSGs were differentially expressed in normal tissues and tumor tissues. The difference in expression was validated based on GEPIA (Figure 5). In addition, based on TCGA UCEC data, we detected eight differentially methylated genes including *EDNRB, CDO1, NDN, PLCD1, ROR2, ESPL1, PRAME, and PTTG1* (Figure 6). Immunohistochemistry results showed that these genes were dysregulated in EC samples. The expression levels of *ENDRB, ROR2, and PLCD1* were lower in EC tissue than in normal tissue, whereas the expression levels of *ESPL1, PRAME, and PTTG1* were higher in EC tissue than in normal tissue. Besides, the expression of *CDO1* showed no difference between normal tissue and tumor tissue (Figure 7). Moreover, ROC curve analysis using “pROC” packages was performed to calculate the capacity of eight genes to distinguish EC tissue from healthy tissue. *EDNRB, CDO1, NDN, ESPRL1, PRAME, and PTTG1* all exhibited excellent diagnostic efficiency (AUC > 0.9), and this efficiency was more obvious when the eight were combinedly used (AUC 0.987) (Figure 8A).

Furthermore, to evaluate the prognostic significance of the six methylated DEGs, we loaded the survival time and gene expression levels from the Human Protein Atlas database. Kaplan‐Meier method was applied to estimate the survival time predicted by each gene. The analysis results showed that a shorter survival was correlated with the lower expression levels of *PLCD1 and ROR2* and the higher expression levels of ESPL1 and PTTG1 (Figure S2).

### 3.6 Gene set enrichment analysis (GSEA)

GSEA was for searching KEGG pathways based on the TCGA samples in order to screen out the potential function of eight genes in EC. The gene sets (n = 546) were enriched in 10 pathways: “hedgehog signaling pathway,” “basal cell carcinoma,” “adipocytokine signaling pathway,” “colorectal cancer,” “oxidative phosphorylation,” “metabolism of xenobiotics by cytochrome p450,” “ribosom,” “antigen processing and presentation,” “systemic lupus erythematosus,” and “olfactory transduction” (Figure 8B‐C) (adj. *P* < .05).

### 3.7 Prognostic signature

**FIGURE 9:**
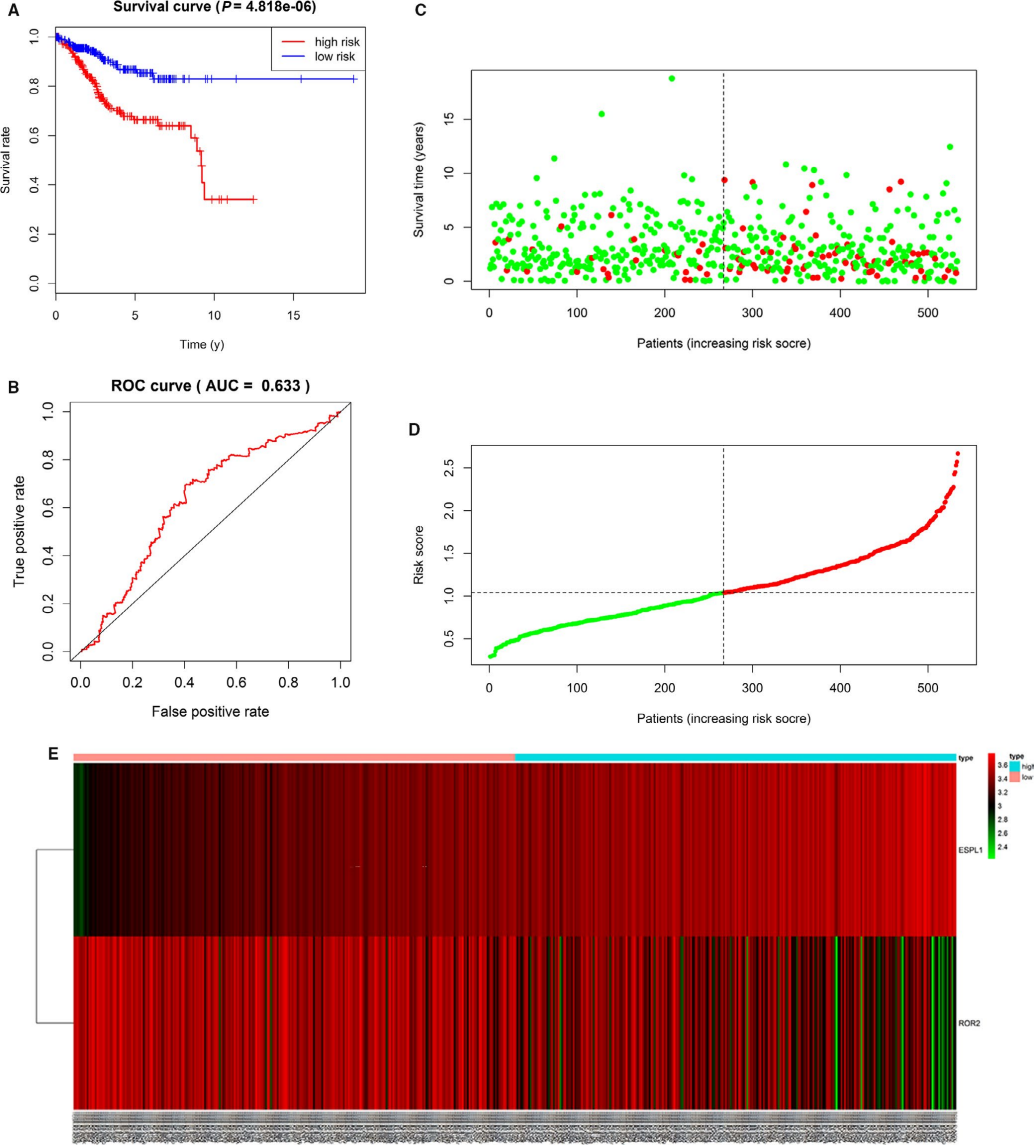
A, Kaplan‐Meier survival analysis, risk score for overall survival. B, ROC curve for predicting 5‐year survival based on risk score. C‐E, The distributions of the two—gene signature, survival status, and expression profiles of the two genes of patients in the TCGA dataset

Univariate Cox proportional hazards regression analyses were performed for the above eight DEGs, including *ESPL1*, *NDN, ROR2, and PLCD1* (Table 1). Multivariate Cox proportional hazards regression analysis was further performed on the above genes, which screened *ROR2* and *ESPL1* (Figure S1). The risk score for predicting overall survival was calculated as follows: Risk score = 0.336* *ESPL1*‐0.101* *ROR2.* According to the median risk score, EC patients were divided into low‐risk (n = 267) and high‐risk (n = 267) groups. Survival analysis showed that low‐risk patients had longer overall survival than high‐risk patients in TCGA cohort (Figure 9A). We also used optimal cutoffs to analyze the prognosis of high‐ and low‐risk groups (*P* < .001) (Figure S4B) In ROC curve analysis, the AUC value for 5‐year survival showed the highest specificity and sensitivity when the risk score was 0.633 (95% confidence interval[CI] 0.52‐0.74) (Figure 9B). *C‐index* = 0.63 (95%CI 0.57‐ 0.69), *P‐value* = 7.06e‐06. As shown in supporting Figure 4B‐C, when clinical factors (including age, stage, grade, and histological type) were combined, the AUC value increased to 0.792. The survival status, the expression of five genes and distribution of risk score in each patient were also analyzed (Figure 9C‐E). In addition, the heatmap showed the expression of the two genes in low‐ and high‐risk patients in the TCGA dataset. We observed significant differences in tumor status (*P* < .001), grade (*P* < .001), histological type (*P* < .001), stage (*P* < .001), and age (*P* < .001) between the high‐ and low‐risk groups (Figure S3).

**TABLE 1:** Univariate Cox proportional hazards regression analysis

Gene	HR	CI	Z	*P*‐value
*ESPL1*	1.429	1.155‐1.769	3.286	.00101
NDN	0.844	0.719‐0.991	−2.061	.0392
ROR2	0.879	0.776‐0.995	−2.037	.0416
PLCD1	0.761	0.581‐0.996	−1.986	.0470

### 3.8 Related small molecule drugs screening

**FIGURE 10:**
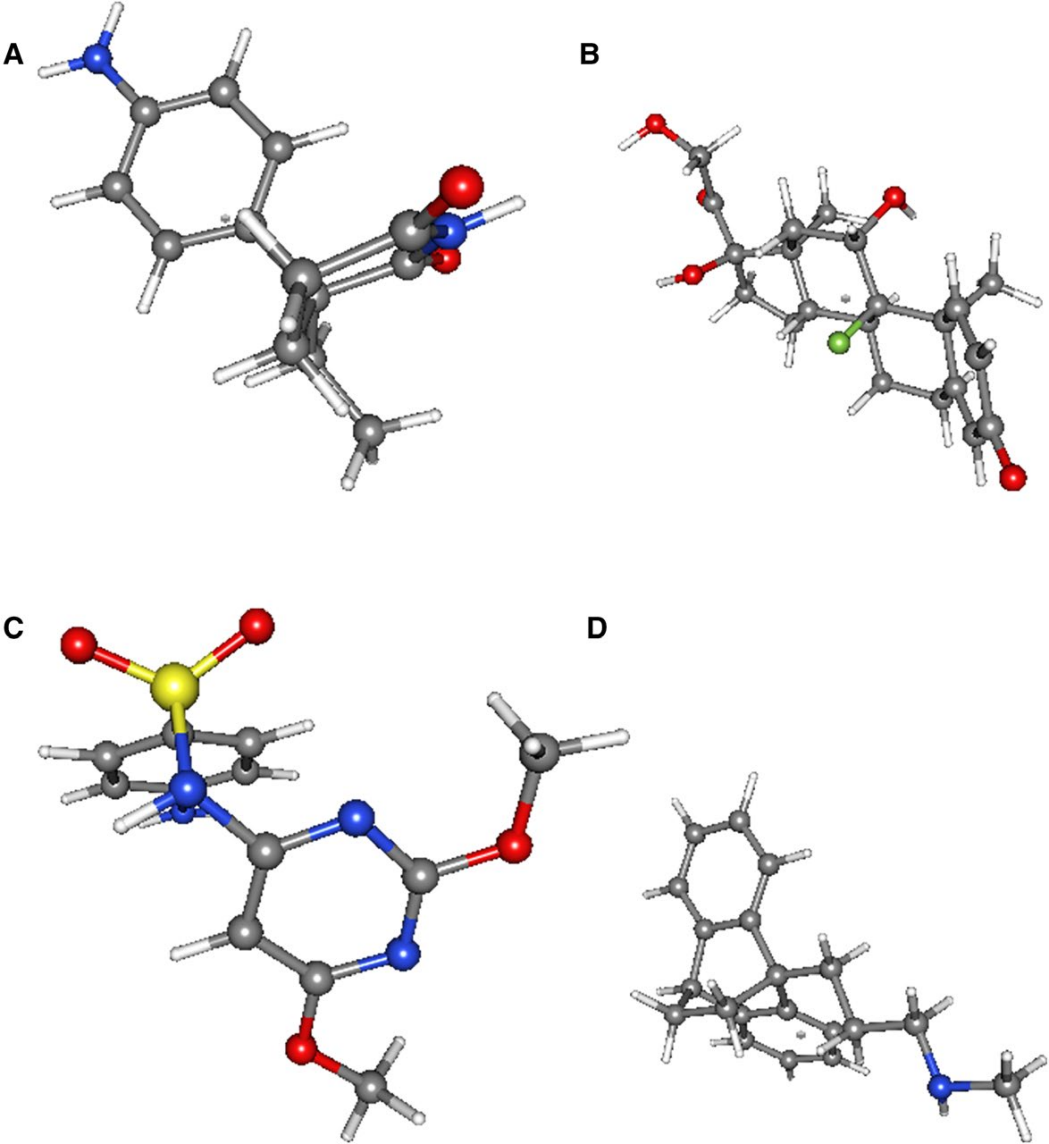
Structures of the top 4 molecule drugs. A, aminoglutethimide B, isoflupredone C, sulfadimethoxine D, maprotiline

To screen out small molecule drugs, we analyzed upregulated genes and hypomethylated genes, as well as downregulated genes and hypermethylated genes with CMap. The top 10 EC‐related small molecules are displayed in Table 2. Among these small molecules, aminoglutethimide and luteolin showed a highly negative correlation with EC. Sulfadimethoxine, maprotiline, isoflupredone, vancomycin, 3‐acetamidocoumarin, clofazimine, adiphenine, and merbromin showed a highly positive correlation with EC. They all might have the potential therapeutic effects on EC. The tomographes of the top 4 potential molecule drugs were researched from Pubchem and shown in Figure 10A‐D.

**TABLE 2:** Results of CMap analysis

Rank	CMap name	Mean	N	Enrichment	*P*‐value
1	Sulfadimethoxine	0.668	5	0.916	.00004
2	Maprotiline	0.605	4	0.899	.0001
3	Isoflupredone	0.752	3	0.944	.0002
4	Aminoglutethimide	−0.718	3	−0.929	.0005
5	Vancomycin	0.621	4	0.848	.0007
6	Luteolin	−0.709	4	−0.85	.0009
7	3‐acetamidocoumarin	0.609	4	0.841	.001
8	Clofazimine	0.483	5	0.783	.001
9	Adiphenine	0.608	5	0.763	.001
10	Merbromin	0.452	5	0.762	.001

## 4 DISCUSSION

EC mortality has increased by more than 100% during the past 20 years.^2^ Only 20% of EC patients are diagnosed before menopause.^28^ Database‐based bioinformatic analysis helps to screen out target biological molecules for early EC diagnosis.

Based on http://www.ncbi.nlm.nih.gov/geo/query/acc.cgi?acc=GSE17025, we extracted the expression matrices of 91 endometrial cancer samples and 12 serous samples of postmenopausal atrophic endometrium. We obtained 1737 DEGs, including 690 upregulated and 1047 downregulated. A total of 4097 DMGs were obtained from http://www.ncbi.nlm.nih.gov/geo/query/acc.cgi?acc=GSE40032. Then, 1761 hypermethylated genes and 2336 hypomethylated genes were retrieved.

With Venn analysis, we detected the overlapped expression between the upregulated genes and hypomethylated genes, as well as downregulated genes and hypermethylated genes. We accessed 121 downregulated hypermethylated genes and 84 upregulated hypomethylated genes. Upregulated hypomethylated genes were overlapped with oncogenes, and downregulated hypermethylated genes with TSGs. Correspondingly, we retrieved seven upregulated hypomethylated oncogenes and 12 downregulated hypermethylated TSGs.

Immune response is involved in tumor development.^29^ This involvement arises with DNA methylation.^30^ Aromatic compound is engaged in the development of lung cancer.^31^ Wnt Signaling is a star pathway associated with many tumors.^32^, ^33^ In the present research, the upregulated hypomethylated genes were mostly enriched in term “immune response” according to GO analysis and in Gastric Cancer Network 1 according to KEGG analysis. The downregulated hypermethylated genes were mainly enriched in aromatic compound catabolic process according to GO analysis and in Wnt Signaling according to KEGG analysis. Our research verified the significance of these genes in EC research.

Downregulated hypermethylated TSGs and upregulated hypomethylated oncogenes, along with their associated genes, conjured up a PPI network with FunRich tool. In this network, the function of the downregulated hypermethylated TSGs and their associated genes were mostly enriched in “pathways in cancer;” the function of the upregulated hypomethylated oncogenes and their associated genes were mostly enriched in “cell cycle.” Cell cycle participates in EC growth and proliferation.^34^ Qiu H et al found that JQ1 suppressed tumor growth via *PTEN/PI3K/AKT* pathway in endometrial cancer.^35^ These all indicated that our functional analysis had guiding significance for EC.

We used GEPIA to confirm the expression and methylation of the 19 selected genes in carcinogenesis. Five upregulated hypomethylated oncogenes, along with 10 downregulated hypermethylated TSGs, were differentially expressed between normal tissue and tumor tissue. Using TCGA UCEC data, we further detected eight DMGs, including *EDNRB, CDO1, NDN, PLCD1, ROR2, ESPL1, PRAME, and PTTG1*. The results of boxplots based on TCGA database were consistent with those from GEO analysis. We next used immunohistochemistry staining to validate these deregulated genes. Finally, ROC curve analysis was for evaluating the capacity of eight genes to distinguish EC tissue from healthy tissue. Univariate Cox proportional hazards regression analysis screened out the genes of significant prognostic value, including *ESPL1*, *NDN, ROR2, and PLCD1*. Multivariate Cox proportional hazards regression analysis screened out ROR2 and *ESPL1*. We calculated the AUC value by combining only the gene expression and not the clinical factors, so the AUC value (0.633) was not high. However, when combined with clinical factors (including age, stage, grade, and histological type), the AUC value increased to 0.792. These hub genes are related to tumor progression. Ror2, a member of the Ror family of receptor tyrosine kinases, acts as a receptor for Wnt5a1. Wnt5a/Ror2 signaling activates the ß‐catenin‐independent noncanonical Wnt pathways.^36^ Michiru Nishita et al proved that Ror2 signaling was involved in tumor invasion.^37^ Yang CM et al further demonstrated that *ROR2* was involved in multiple biological behaviors of renal carcinoma.^38^ Equally, *ESPL1* was proven to be a cancer oncogene for breast cancer.^39^ Ushiku H et al demonstrated that *CDO1* promoted DNA methylation in the process of gastric cancer.^40^ Ushiku H et al also demonstrated that promoter DNA methylation of *CDO1* gene regulated esophageal squamous cell carcinoma progression.^41^ *CDO1* affected the procession of prostate cancer,^42^ clear‐cell renal cell cancer,^43^ breast cancer,^44^ and lung cancer.^45^ Hu YH et al found that hypermethylation of *NDN* promoted the cell proliferation by activating the Wnt signaling pathway in colorectal cancer.^46^ Yang H et al found that NDN inhibited ovarian cancer development.^47^ Phospholipase C δ1 (*PLCD1*) manipulates the biological behaviors of pancreatic cancer cells.^48^ Methylation of *PLCD1* can be regulated to improve the treatment of breast cancer.^49^ Epigenetic inactivation of *PLCD1* occurs in chronic myeloid leukemia.^50^ PRAME is implicated in the growth and metastasis of breast cancer, the hypomethylation of epithelial ovarian cancer, and the prognosis of nonsmall cell lung cancer.^51^ PTTG1, as an androgen responsive gene, acts in the progression of androgen‐induced prostate cancer, colorectal cancer, breast cancer, ovarian cancer, and bladder cancer.^52^, ^53^, ^54^, ^55^ Endothelin receptor type B (EDNRB) gene, a member of G protein‐coupled receptor superfamily, plays in the development of embryonic and enteric ganglia.^56^ Reza Mousavi Ardehaie et al demonstrated aberrantly methylated EDNRB acted as a potential diagnostic biomarker in sporadic colorectal cancer.^57^ However, these genes have not been linked to EC. Here we demonstrate that their methylation may be involved in the development of EC.

CMap showed EC had negative correlation with aminoglutethimide and luteolin and positive correlation with sulfadimethoxine, maprotiline, isoflupredone, vancomycin, 3‐acetamidocoumarin, clofazimine, adiphenine, and merbromin. Aminoglutethimide has a therapeutic effect on breast cancer and luteolin on digestive tumors.^58^, ^59^ Research by Liu XS et al suggested that aminoglutethimide was effective in treating EC through improving endocrine environments and inhibiting cell growth.^60^ Sulfadimethoxine has been used in the treatment of bladder cancer.^61^ Hsu SS et al found antidepressant maprotiline worked in Ca^2+^ movement and the proliferation of human prostate cancer cells.^62^ The mentioned results all suggested that the drugs screened by *C*_map_ were useful in fighting against EC.

However, this study has some limitations. Firstly, although an internal verification of the potential aberrantly methylated DEGs and dysregulated pathways was performed, a multicenter and prospective study is needed to evaluate the practicality of eight hub genes. Secondly, further in vivo and in vitro experimental verification is also needed to elucidate the molecular mechanisms. Finally, due to the lack of clinical information from an external database (such as GEO), the prognostic value of our signature should be further warranted.

## 5 CONCLUSION

This research inferred potential aberrantly methylated DEGs and dysregulated pathways may participate in EC development and firstly reported eight hub genes including *EDNRB, CDO1, NDN, PLCD1, ROR2, ESPL1, PRAME, and PTTG1* that could be used to predict EC prognosis. Aminoglutethimide and luteolin may be used to fight against EC.

## CONFLICT OF INTERESTS

The authors report no conflict of interest in this work.

## AUTHOR CONTRIBUTION

Liu Jinhui was involved in data curation, formal analysis, and methodology. 

Cheng Wenjun was involved in funding acquisition. Wan Yicong, Li Siyue, Jiang Yi, Ma Xiaoling, and Zhou Shulin were involved in supervision. Li Siyue and Qiu Huaide were involved in writing—original draft. Li Siyue was involved in writing—review and editing.

## ETHICS APPROVAL AND CONSENT TO PARTICIPATE

Not applicable.

## CONSENT FOR PUBLICATION

Written informed consent for publication was obtained from all participants.

## Supporting information

Fig S1

Fig S2

Fig S3

Fig S4

Table S1

Table S2

Fig S1‐S4

## Data Availability

All the data in this study are available from GEO database and TCGA database.

## References

[1] Liu C , Zhang YH , Deng Q , et al. Cancer‐related triplets of mRNA‐lncRNA‐miRNA revealed by integrative network in uterine corpus endometrial carcinoma. Biomed Res Int. 2017;2017:3859582. 28280730 10.1155/2017/3859582 PMC5320387

[2] Braun MM , Overbeek‐Wager EA , Grumbo RJ . Diagnosis and management of endometrial cancer. Am Fam Physician. 2016;93:468‐474. 26977831

[3] Neppe C , Land R , Obermair A . Wrigley forceps to deliver a bulky uterus following a total laparoscopic hysterectomy for endometrial cancer. Aust N Z J Obstet Gynaecol. 2005;45:444‐445. 16171485 10.1111/j.1479-828X.2005.00460.x

[4] Yin L , Cai Z , Zhu B , Xu C . Identification of key pathways and genes in the dynamic progression of HCC based on WGCNA. Genes (Basel). 2018;9(2):92. 10.3390/genes9020092 PMC5852588 29443924

[5] Yao S , Liu T . Analysis of differential gene expression caused by cervical intraepithelial neoplasia based on GEO database. Oncol Lett. 2018;15:8319‐8324. 29805564 10.3892/ol.2018.8403 PMC5950031

[6] Liu X , Hu AX , Zhao JL , Chen FL . Identification of key gene modules in human osteosarcoma by co‐expression analysis weighted gene co‐expression network analysis (WGCNA). J Cell Biochem. 2017;118:3953‐3959. 28398605 10.1002/jcb.26050

[7] Zhang H , Gelernter J . Review: DNA methylation and alcohol use disorders: progress and challenges. Am J Addict. 2017;26:502‐515. 27759945 10.1111/ajad.12465 PMC6003819

[8] Jablonska E , Reszka E . Selenium and epigenetics in cancer: focus on DNA methylation. Adv Cancer Res. 2017;136:193‐234. 29054419 10.1016/bs.acr.2017.07.002

[9] Shridhar K , Walia GK , Aggarwal A , et al. DNA methylation markers for oral pre‐cancer progression: a critical review. Oral Oncol. 2016;53:1‐9. 26690652 10.1016/j.oraloncology.2015.11.012 PMC4788701

[10] Bhat S , Kabekkodu SP , Varghese VK , et al. Aberrant gene‐specific DNA methylation signature analysis in cervical cancer. Tumour Biol. 2017;39:1010428317694573. 28351298 10.1177/1010428317694573

[11] Huang RL , Su PH , Liao YP , et al. Integrated epigenomics analysis reveals a DNA methylation panel for endometrial cancer detection using cervical scrapings. Clin Cancer Res. 2017;23:263‐272. 27507616 10.1158/1078-0432.CCR-16-0863

[12] Chang CC , Wang HC , Liao YP , et al. The feasibility of detecting endometrial and ovarian cancer using DNA methylation biomarkers in cervical scrapings. J Gynecol Oncol. 2018;29:e17. 29185275 10.3802/jgo.2018.29.e17 PMC5709527

[13] Fialkova V , Vidomanova E , Balharek T , et al. DNA methylation as mechanism of apoptotic resistance development in endometrial cancer patients. Gen Physiol Biophys. 2017;36:521‐529. 29372685 10.4149/gpb_2017032

[14] Caplakova V , Babusikova E , Blahovcova E , Balharek T , Zelieskova M , Hatok J . DNA methylation machinery in the endometrium and endometrial cancer. Anticancer Res. 2016;36:4407‐4420. 27630276 10.21873/anticanres.10984

[15] Gautier L , Cope L , Bolstad BM , Irizarry RA . affy–analysis of Affymetrix GeneChip data at the probe level. Bioinformatics. 2004;20:307‐315. 14960456 10.1093/bioinformatics/btg405

[16] Tian Y , Morris TJ , Webster AP , et al. ChAMP: updated methylation analysis pipeline for Illumina BeadChips. Bioinformatics. 2017;33:3982‐3984. 28961746 10.1093/bioinformatics/btx513 PMC5860089

[17] Ritchie ME , Phipson B , Wu D , et al. limma powers differential expression analyses for RNA‐sequencing and microarray studies. Nucleic Acids Res. 2015;43:e47. 25605792 10.1093/nar/gkv007 PMC4402510

[18] Wu K , Yin X , Jin Y , Liu F , Gao J . Identification of aberrantly methylated differentially expressed genes in prostate carcinoma using integrated bioinformatics. Cancer Cell Int. 2019;19:51. 30872976 10.1186/s12935-019-0763-8 PMC6402097

[19] Zhang X , Feng H , Li D , Liu S , Amizuka N , Li M . Identification of differentially expressed genes induced by aberrant methylation in oral squamous cell carcinomas using integrated bioinformatic analysis. Int J Mol Sci. 2018;19(6):1698. 10.3390/ijms19061698 PMC6032197 29875348

[20] Zhang M , Lv X , Jiang Y , Li G , Qiao Q . Identification of aberrantly methylated differentially expressed genes in glioblastoma multiforme and their association with patient survival. Exp Ther Med. 2019;18:2140‐2152. 31452706 10.3892/etm.2019.7807 PMC6704589

[21] Chen H , Boutros PC . VennDiagram: a package for the generation of highly‐customizable Venn and Euler diagrams in R. BMC Bioinformatics. 2011;12:35. 21269502 10.1186/1471-2105-12-35 PMC3041657

[22] Pathan M , Keerthikumar S , Ang CS , et al. FunRich: an open access standalone functional enrichment and interaction network analysis tool. Proteomics. 2015;15:2597‐2601. 25921073 10.1002/pmic.201400515

[23] Szklarczyk D , Franceschini A , Wyder S , et al. STRING v10: protein‐protein interaction networks, integrated over the tree of life. Nucleic Acids Res. 2015;43:D447‐D452. 25352553 10.1093/nar/gku1003 PMC4383874

[24] Tang Z , Li C , Kang B , Gao G , Li C , Zhang Z . GEPIA: a web server for cancer and normal gene expression profiling and interactive analyses. Nucleic Acids Res. 2017;45:W98‐w102. 28407145 10.1093/nar/gkx247 PMC5570223

[25] Pencina MJ , D'Agostino RB . Overall C as a measure of discrimination in survival analysis: model specific population value and confidence interval estimation. Stat Med. 2004;23:2109‐2123. 15211606 10.1002/sim.1802

[26] Mori A , Yamashita S , Nakajima M , et al. CMAP decrement as a potential diagnostic marker for ALS. Acta Neurol Scand. 2016;134:49–53. 26434688 10.1111/ane.12510

[27] Subramanian A , Kuehn H , Gould J , Tamayo P , Mesirov JP . GSEA‐P: a desktop application for gene set enrichment analysis. Bioinformatics. 2007;23:3251‐3253. 17644558 10.1093/bioinformatics/btm369

[28] Sorosky JI . Endometrial cancer. Obstet Gynecol. 2012;120:383‐397. 22825101 10.1097/AOG.0b013e3182605bf1

[29] Bibeau F , Bazille C , Svrcek M , et al. Immune response and digestive cancers: prognostic and therapeutic implications. Ann Pathol. 2017;37:111‐116. 28111038 10.1016/j.annpat.2016.12.008

[30] Jeschke J , Bizet M , Desmedt C , et al. DNA methylation‐based immune response signature improves patient diagnosis in multiple cancers. J Clin Invest. 2017;127:3090‐3102. 28714863 10.1172/JCI91095 PMC5531413

[31] Moorthy B , Chu C , Carlin DJ . Polycyclic aromatic hydrocarbons: from metabolism to lung cancer. Toxicol Sci. 2015;145:5‐15. 25911656 10.1093/toxsci/kfv040 PMC4408964

[32] Schneider JA , Logan SK . Revisiting the role of Wnt/beta‐catenin signaling in prostate cancer. Mol Cell Endocrinol. 2018;462:3‐8. 28189566 10.1016/j.mce.2017.02.008 PMC5550366

[33] Yang J , Chen J , He J , et al. Wnt signaling as potential therapeutic target in lung cancer. Expert Opin Ther Targets. 2016;20:999‐1015. 26882052 10.1517/14728222.2016.1154945

[34] Zhou Y , Wang K , Zhen S , Wang R , Luo W . Carfilzomib induces G2/M cell cycle arrest in human endometrial cancer cells via upregulation of p21(Waf1/Cip1) and p27(Kip1). Taiwan J Obstet Gynecol. 2016;55:847‐851. 28040131 10.1016/j.tjog.2016.09.003

[35] Qiu H , Li J , Clark LH , et al. JQ1 suppresses tumor growth via PTEN/PI3K/AKT pathway in endometrial cancer. Oncotarget. 2016;7:66809‐66821. 27572308 10.18632/oncotarget.11631 PMC5341839

[36] Oishi I , Suzuki H , Onishi N , et al. The receptor tyrosine kinase Ror2 is involved in non‐canonical Wnt5a/JNK signalling pathway. Genes Cells. 2003;8:645‐654. 12839624 10.1046/j.1365-2443.2003.00662.x

[37] Nishita M , Park SY , Nishio T , et al. Ror2 signaling regulates Golgi structure and transport through IFT20 for tumor invasiveness. Sci Rep. 2017;7:1. 28127051 10.1038/s41598-016-0028-x PMC5428335

[38] Yang CM , Ji S , Li Y , Fu LY , Jiang T , Meng FD . Ror2, a developmentally regulated kinase, is associated with tumor growth, apoptosis, migration, and invasion in renal cell carcinoma. Oncol Res. 2017;25:195‐205. 28277191 10.3727/096504016X14732772150424 PMC7840799

[39] Finetti P , Guille A , Adelaide J , Birnbaum D , Chaffanet M , Bertucci F . ESPL1 is a candidate oncogene of luminal B breast cancers. Breast Cancer Res Treat. 2014;147:51‐59. 25086634 10.1007/s10549-014-3070-z

[40] Ushiku H , Yamashita K , Ema A , et al. DNA diagnosis of peritoneal fluid cytology test by CDO1 promoter DNA hypermethylation in gastric cancer. Gastric Cancer. 2017;20:784‐792. 28243814 10.1007/s10120-017-0697-6

[41] Ushiku H , Yamashita K , Katoh H , et al. Promoter DNA methylation of CDO1 gene and its clinical significance in esophageal squamous cell carcinoma. Dis Esophagus. 2017;30:1‐9. 10.1111/dote.12496 27629777

[42] Meller S , Zipfel L , Gevensleben H , et al. CDO1 promoter methylation is associated with gene silencing and is a prognostic biomarker for biochemical recurrence‐free survival in prostate cancer patients. Epigenetics. 2016;11:871‐880. 27689475 10.1080/15592294.2016.1241931 PMC5193493

[43] Deckers IA , Schouten LJ , Van Neste L , et al. Promoter methylation of CDO1 identifies clear‐cell Renal cell cancer patients with poor survival outcome. Clin Cancer Res. 2015;21:3492‐3500. 25904753 10.1158/1078-0432.CCR-14-2049 PMC4612631

[44] Minatani N , Waraya M , Yamashita K , et al. Prognostic significance of promoter DNA hypermethylation of cysteine dioxygenase 1 (CDO1) gene in primary breast cancer. PLoS One. 2016;11:e0144862. 26785325 10.1371/journal.pone.0144862 PMC4718689

[45] Wrangle J , Machida EO , Danilova L , et al. Functional identification of cancer‐specific methylation of CDO1, HOXA9, and TAC1 for the diagnosis of lung cancer. Clin Cancer Res. 2014;20:1856‐1864. 24486589 10.1158/1078-0432.CCR-13-2109 PMC4019442

[46] Hu YH , Chen Q , Lu YX , et al. Hypermethylation of NDN promotes cell proliferation by activating the Wnt signaling pathway in colorectal cancer. Oncotarget. 2017;8:46191‐46203. 28521288 10.18632/oncotarget.17580 PMC5542259

[47] Yang H , Das P , Yu Y , et al. NDN is an imprinted tumor suppressor gene that is downregulated in ovarian cancers through genetic and epigenetic mechanisms. Oncotarget. 2016;7:3018‐3032. 26689988 10.18632/oncotarget.6576 PMC4823087

[48] Hu D , Jiang Z . Phospholipase C delta1 (PLCD1) inhibits the proliferation, invasion and migration of CAPAN‐1 and BXPC‐3 pancreatic cancer cells. Xi Bao Yu Fen Zi Mian Yi Xue Za Zhi. 2016;32:739‐745. 27371838

[49] Mu H , Wang N , Zhao L , et al. Methylation of PLCD1 and adenovirus‐mediated PLCD1 overexpression elicits a gene therapy effect on human breast cancer. Exp Cell Res. 2015;332:179‐189. 25655282 10.1016/j.yexcr.2015.01.017

[50] Song JJ , Liu Q , Li Y , et al. Epigenetic inactivation of PLCD1 in chronic myeloid leukemia. Int J Mol Med. 2012;30:179‐184. 22576628 10.3892/ijmm.2012.970

[51] Pan SH , Su KY , Spiessens B , et al. Gene expression of MAGE‐A3 and PRAME tumor antigens and EGFR mutational status in Taiwanese non‐small cell lung cancer patients. Asia Pac J Clin Oncol. 2017;13:e212‐e223. 27519286 10.1111/ajco.12586

[52] Ren Q , Jin B . The clinical value and biological function of PTTG1 in colorectal cancer. Biomed Pharmacother. 2017;89:108‐115. 28219049 10.1016/j.biopha.2017.01.115

[53] Xiea Y , Wangb R . Pttg1 promotes growth of breast cancer through P27 nuclear exclusion. Cell Physiol Biochem. 2016;38:393‐400. 26824458 10.1159/000438660

[54] Nakachi I , Helfrich BA , Spillman MA , et al. PTTG1 levels are predictive of saracatinib sensitivity in ovarian cancer cell lines. Clin Transl Sci. 2016;9:293‐301. 27766744 10.1111/cts.12413 PMC5351005

[55] Xiang W , Wu X , Huang C , et al. PTTG1 regulated by miR‐146a‐3p promotes bladder cancer migration, invasion, metastasis and growth. Oncotarget. 2017;8:664‐678. 27893422 10.18632/oncotarget.13507 PMC5352187

[56] Chen C , Wang L , Liao Q , et al. Hypermethylation of EDNRB promoter contributes to the risk of colorectal cancer. Diagn Pathol. 2013;8:199. 24326135 10.1186/1746-1596-8-199 PMC4029727

[57] Mousavi Ardehaie R , Hashemzadeh S , Behrouz Sharif S , Ghojazadeh M , Teimoori‐Toolabi L , Sakhinia E . Aberrant methylated EDNRB can act as a potential diagnostic biomarker in sporadic colorectal cancer while KISS1 is controversial. Bioengineered. 2017;8:555‐564. 28140749 10.1080/21655979.2017.1283458 PMC5639868

[58] Zang MD , Hu L , Fan ZY , et al. Luteolin suppresses gastric cancer progression by reversing epithelial‐mesenchymal transition via suppression of the Notch signaling pathway. J Transl Med. 2017;15:52. 28241766 10.1186/s12967-017-1151-6 PMC5327575

[59] Liu Y , Lang T , Jin B , et al. Luteolin inhibits colorectal cancer cell epithelial‐to‐mesenchymal transition by suppressing CREB1 expression revealed by comparative proteomics study. J Proteomics. 2017;161:1‐10. 28391045 10.1016/j.jprot.2017.04.005

[60] Liu XS , Zhang X . Comparison between the roles of aminoglutethimide and hydroxyprogesterone caproates in the treatment of endometrial cancer. Zhonghua Fu Chan Ke Za Zhi. 1995;30:479‐482. 8565696

[61] Vila‐Caballer M , Codolo G , Munari F , et al. A pH‐sensitive stearoyl‐PEG‐poly(methacryloyl sulfadimethoxine)‐decorated liposome system for protein delivery: an application for bladder cancer treatment. J Control Release. 2016;238:31‐42. 27444816 10.1016/j.jconrel.2016.07.024

[62] Hsu SS , Chen WC , Lo YK , et al. Effect of the antidepressant maprotiline on Ca^2+^ movement and proliferation in human prostate cancer cells. Clin Exp Pharmacol Physiol. 2004;31:444‐449. 15236632 10.1111/j.1440-1681.2004.04024.x

