# Corrigendum

**DOI:** 10.1002/cam4.3234

**Published:** 2020-06-18

In the article by Liu et al,^1^ the meaning of CMap as listed in the Abstract and list of abbreviations is incorrect. Instead of “Compound muscle action potential,” CMap should be defined as "Connectivity Map." The authors confirm that all of the results and conclusions of the article remain unchanged.

The authors apologize for this error.
