# Supplementary figures and images for: Identification of aberrantly methylated differentially expressed genes and associated pathways in endometrial cancer using integrated bioinformatic analysis
Source: Cancer Med. 2020 Mar 14;9(10):3522–36. doi: 10.1002/cam4.2956 (PMC7221444; doi:10.1002/cam4.2956)

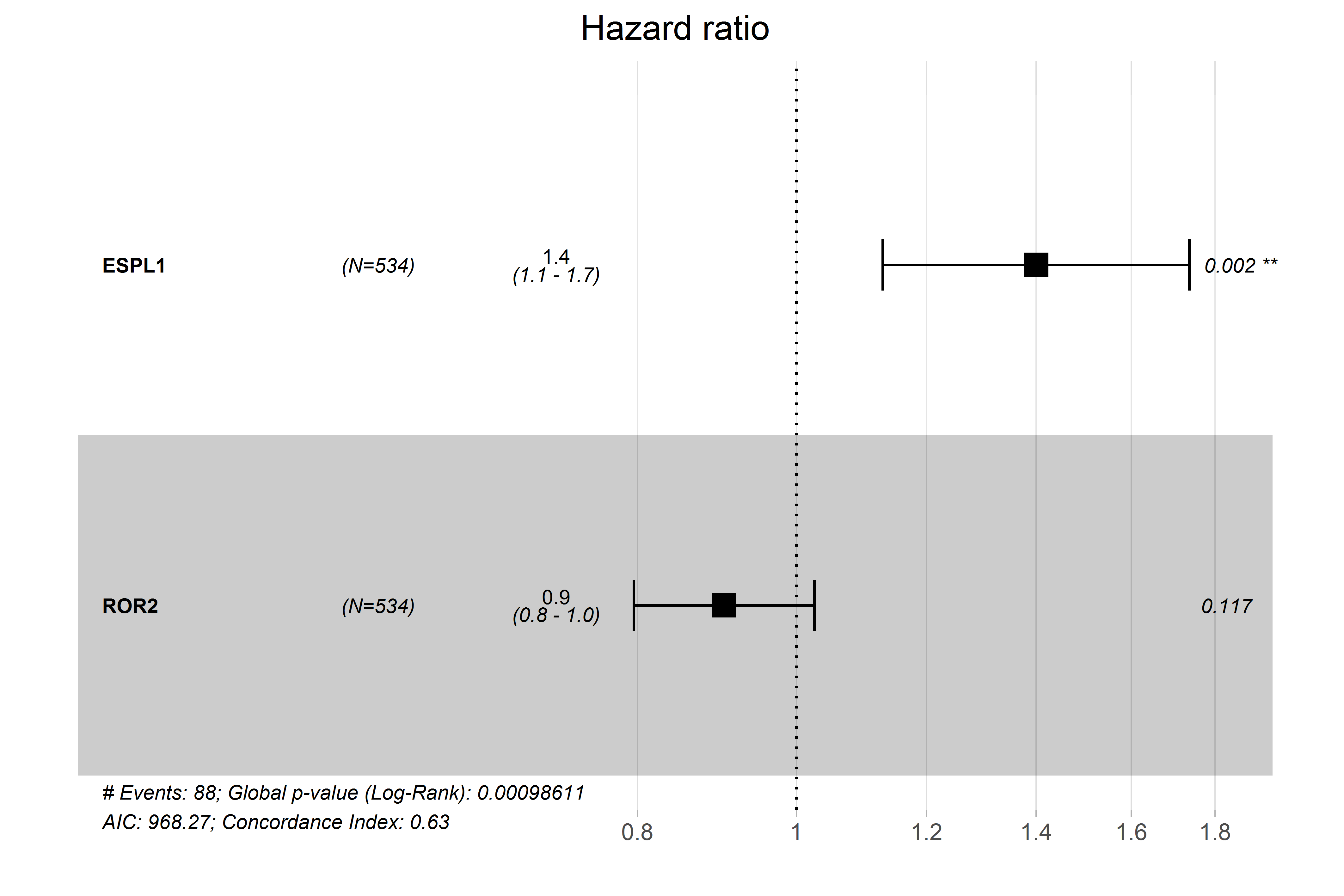

Supplement: Supplementary file 1 — Fig S1 [file CAM4-9-3522-s001.tiff]

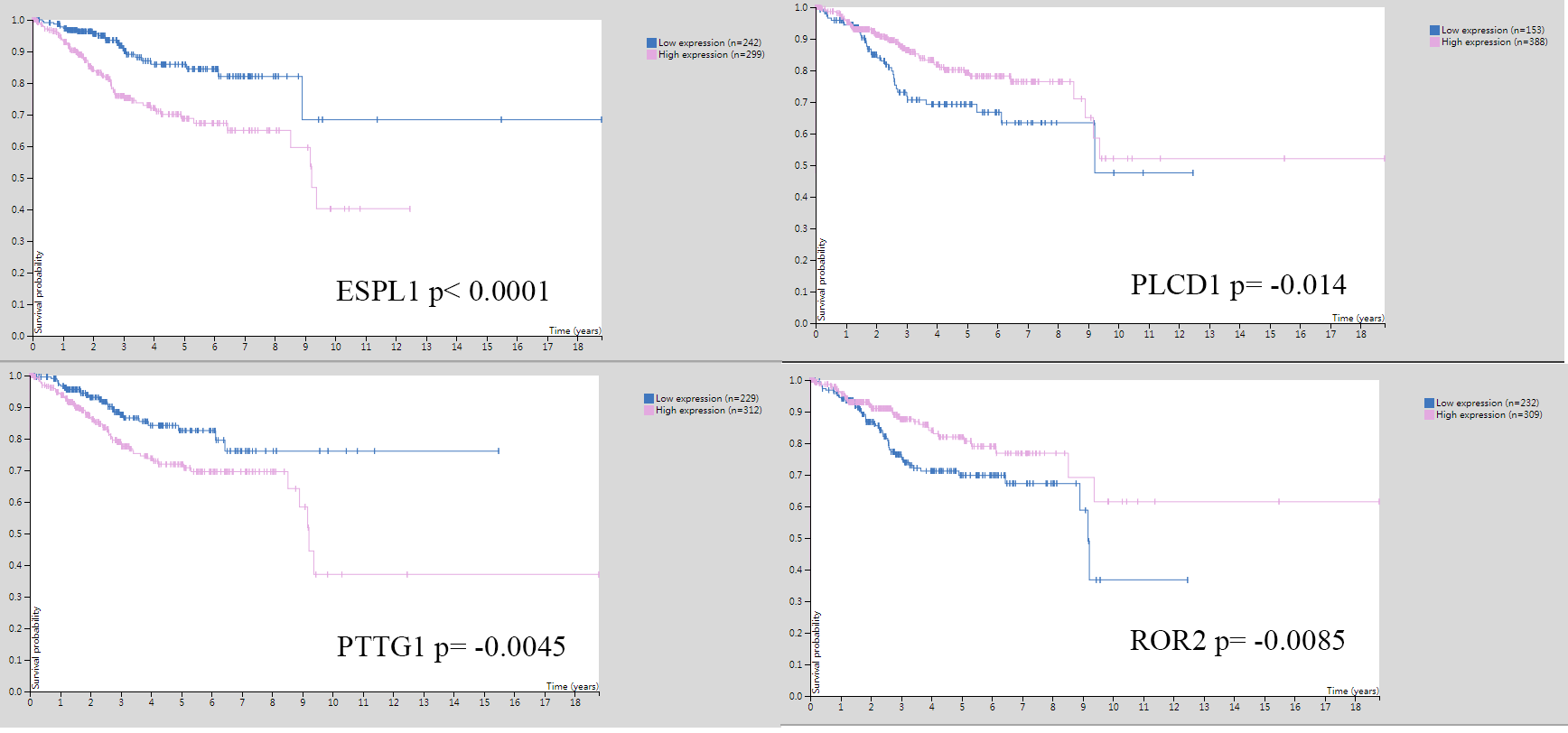

Supplement: Supplementary file 2 — Fig S2 [file CAM4-9-3522-s002.tif]

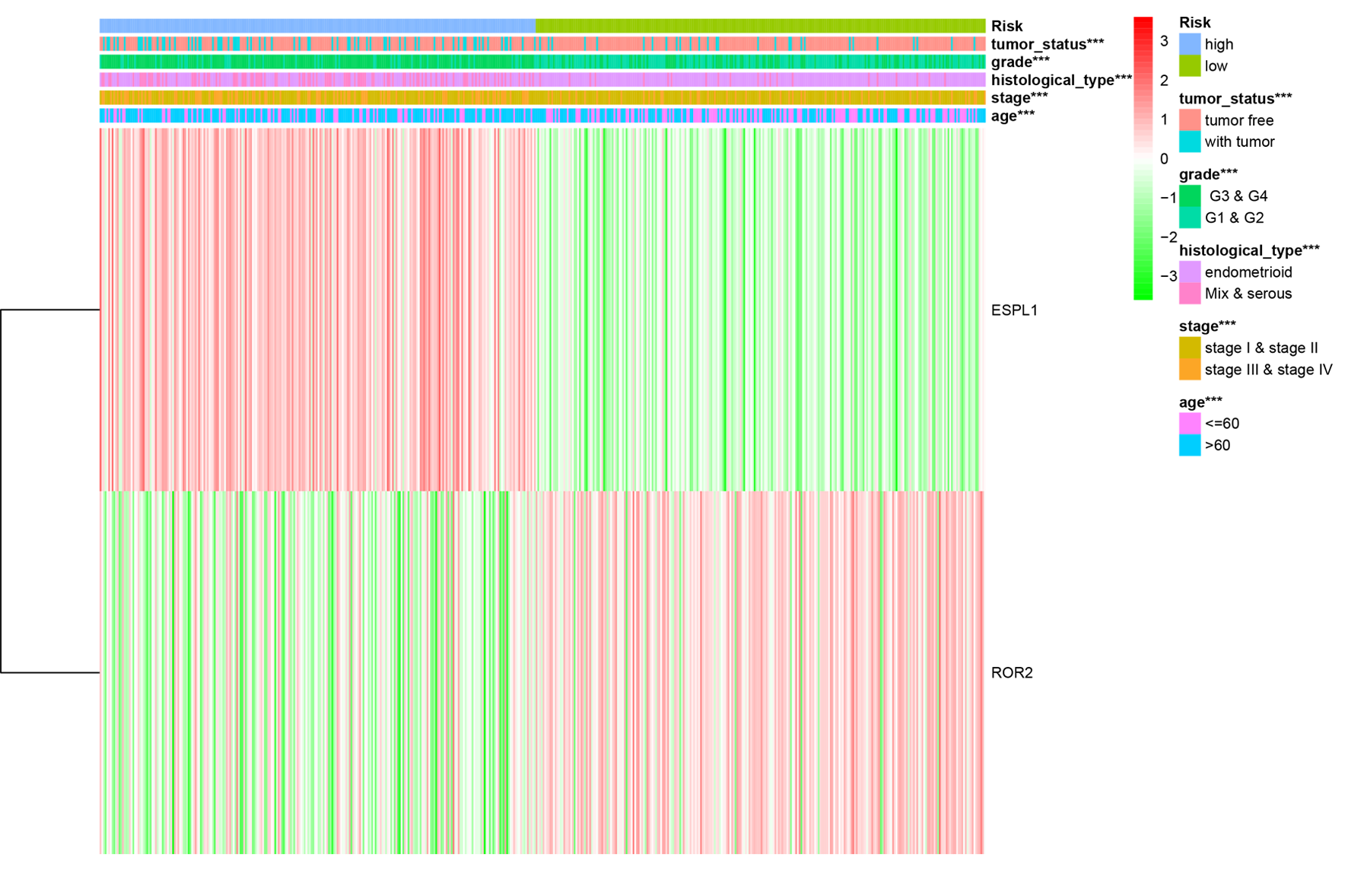

Supplement: Supplementary file 3 — Fig S3 [file CAM4-9-3522-s003.tif]

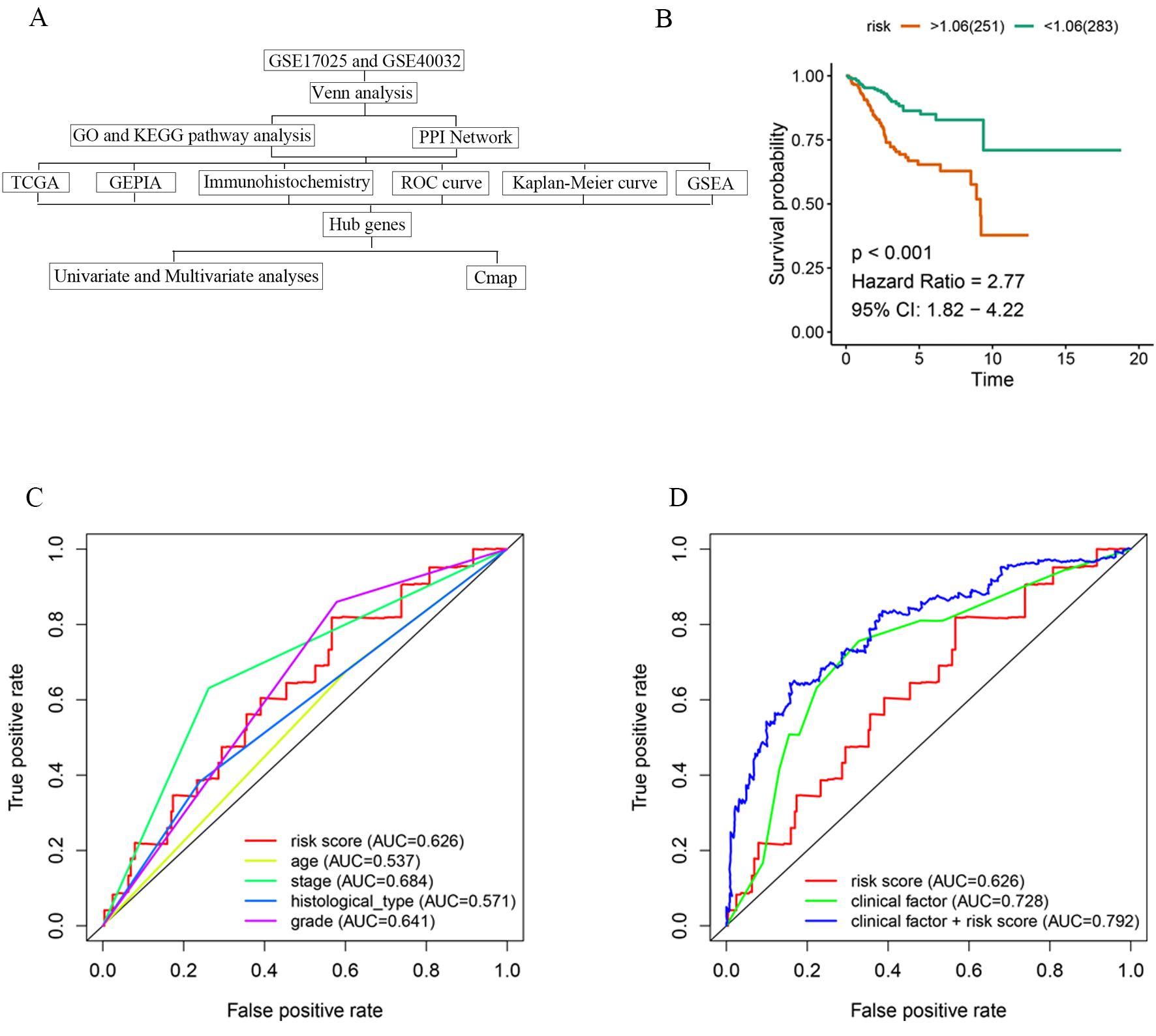

Supplement: Supplementary file 4 — Fig S4 [file CAM4-9-3522-s004.png]
