# Supplementary material for: Identification of aberrantly methylated differentially expressed genes and associated pathways in endometrial cancer using integrated bioinformatic analysis
Source: Cancer Med. 2020 Mar 14;9(10):3522–36. doi: 10.1002/cam4.2956 (PMC7221444; doi:10.1002/cam4.2956)

**Supplementary Figure 1** Multivariate Cox proportional hazards regression analysis screened out four hub genes.

**
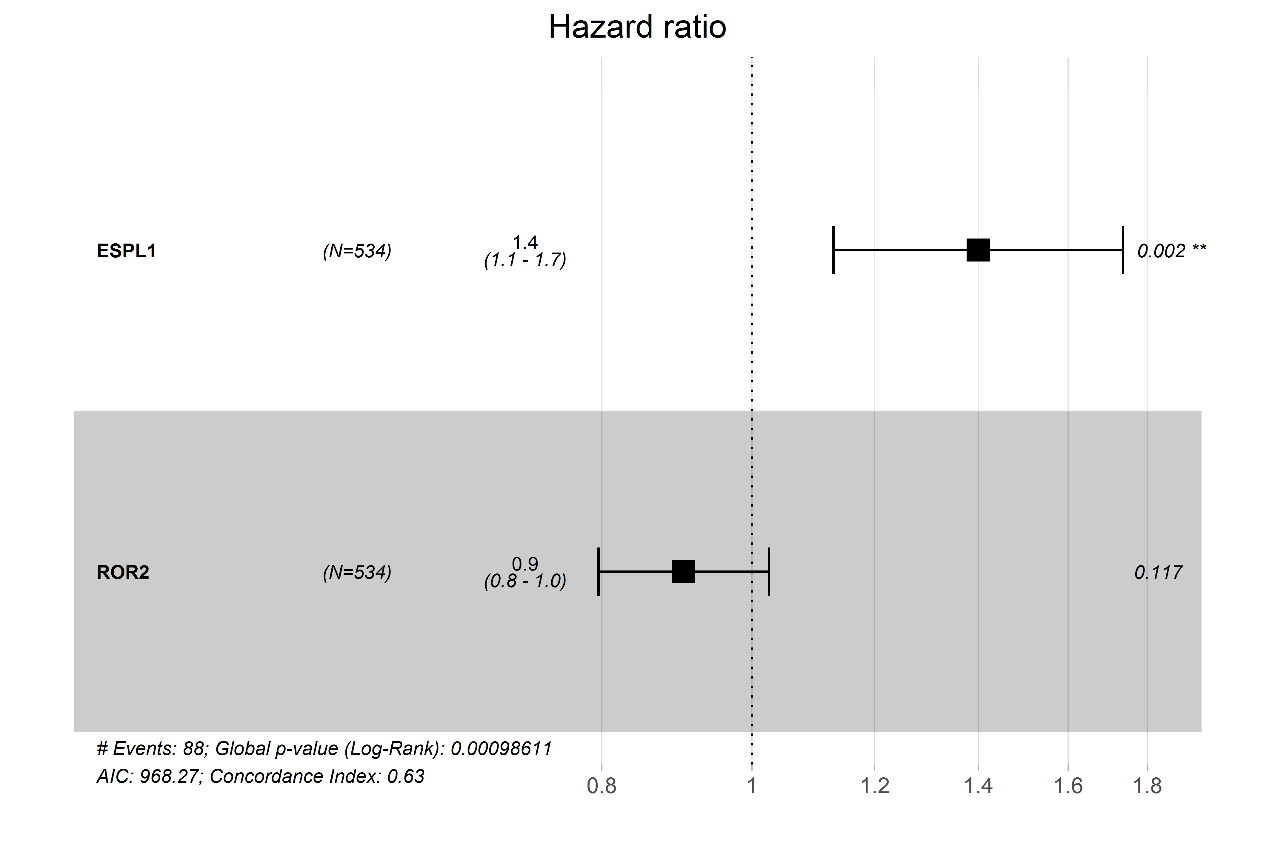
**

**Supplementary Figure 2** The prognostic significance of hub genes in endometrial cancer. Lower levels of PLCD1 and ROR2 were associated with poorer overall survival, and the higher levels of PTTG1 and ESR1 were associated with poorer overall survival.


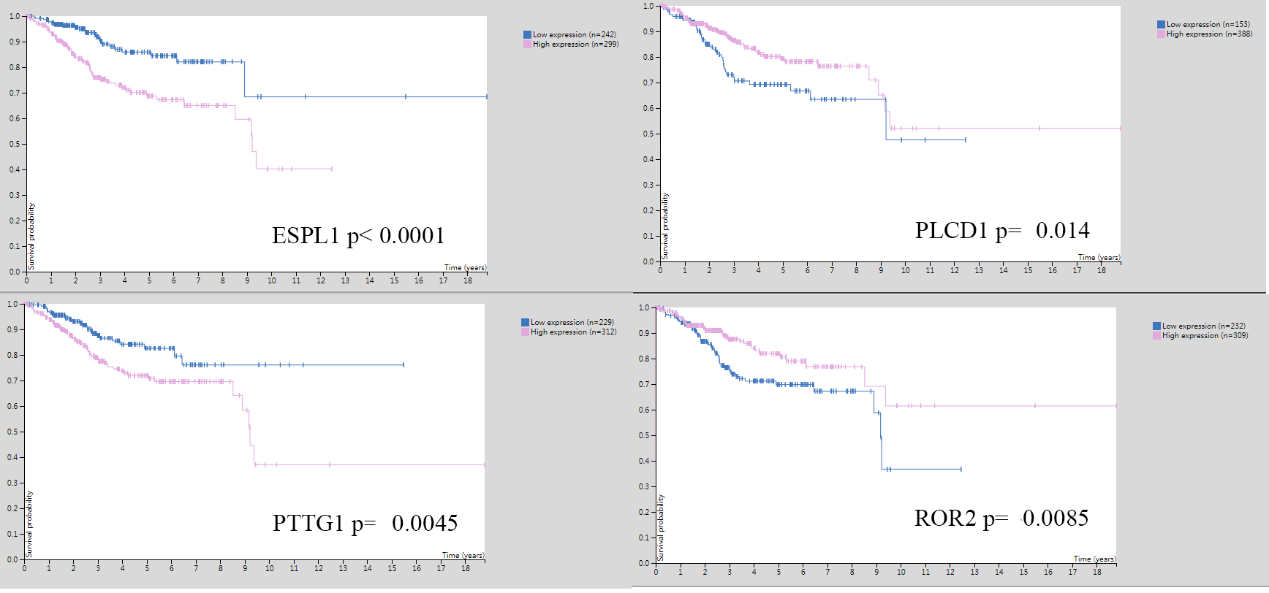


**Supplementary Figure 3** The heatmap showing the distribution of clinicopathological features between the low- and high-risk groups.


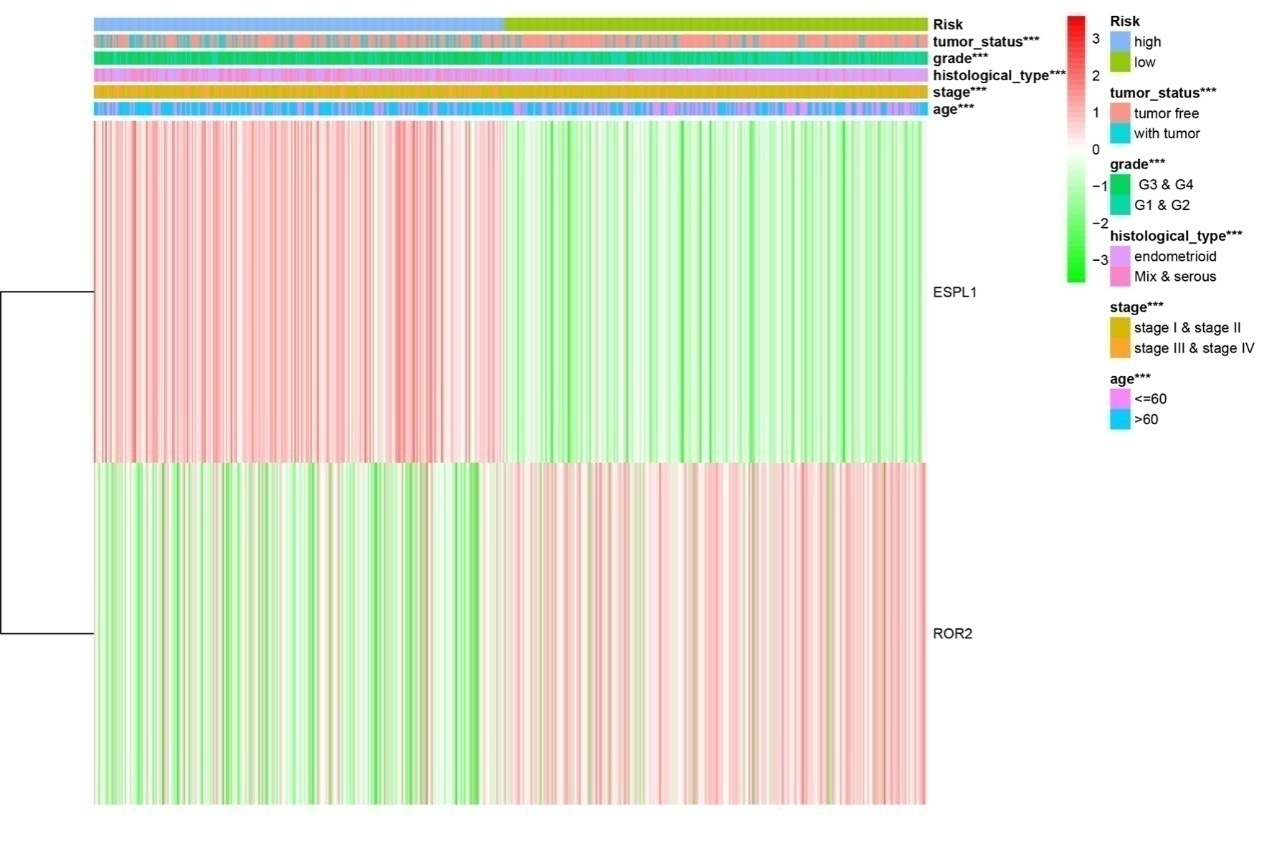


**Supplementary Figure 4** (A) flow chart. (B) survival analysis of the high- and low- risk group patients which were defined by the cut off optimization. (C-D) Time-dependent ROC curve analyses of the riskscore, age ，stage，histological type and grade in TCGA cohort.


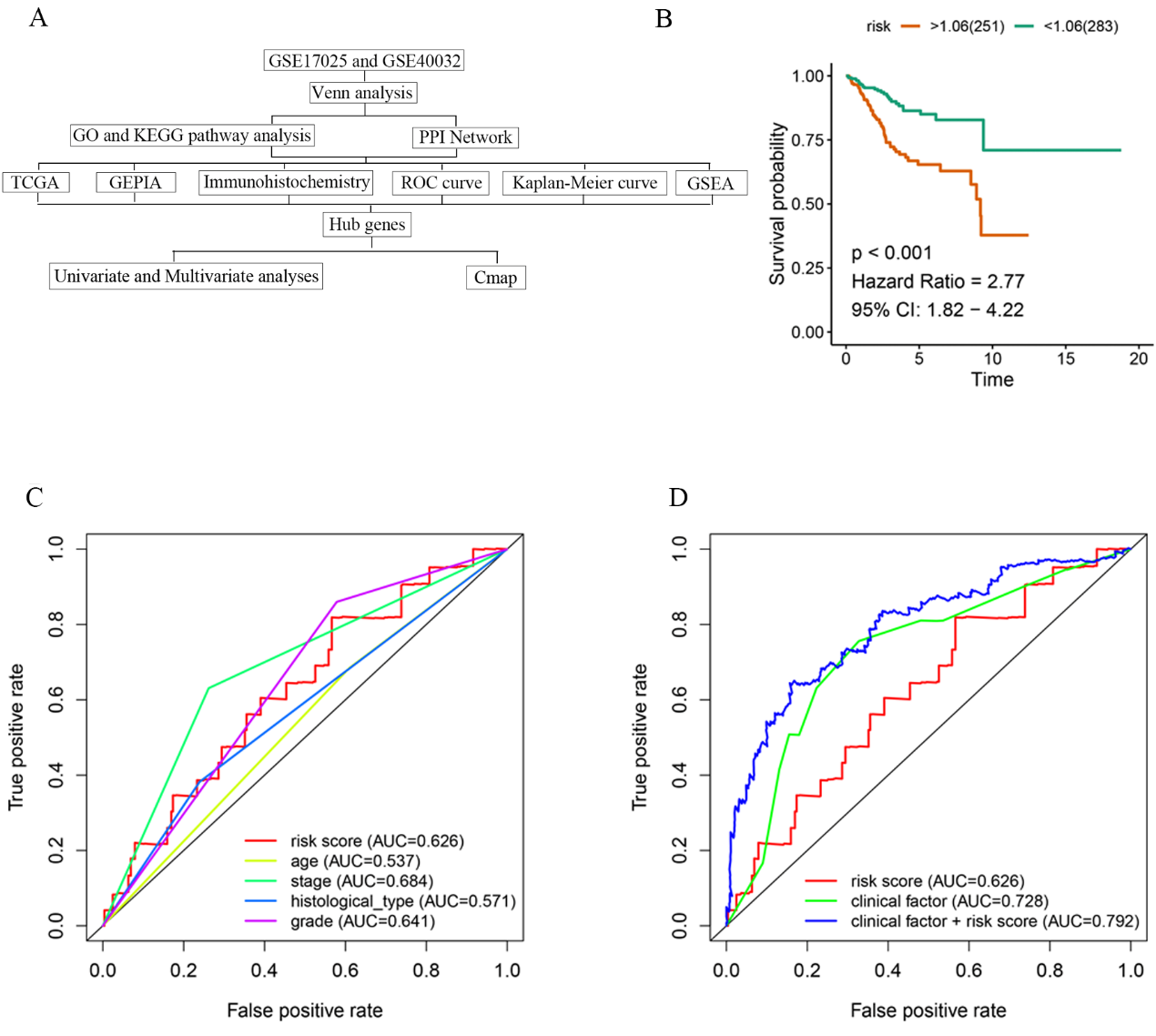

Supplement: Supplementary file 7 — Fig S1‐S4 [file CAM4-9-3522-s007.docx]
